# Personalized targeting of BCL2 family proteins overcomes acquired resistance to BRAF-MEK inhibitors in preclinical melanoma

**DOI:** 10.1038/s41467-026-74691-9

**Published:** 2026-06-26

**Authors:** Y. N. Vashisht Gopal, Evelyn de Groot, Kaley Loftin, Barbara Knighton, Debora Ledesma, Courtney Hudgens, Diana Shamsutdinova, Mehboob Ali, Zhenlin Ju, Min Xiao, Toshitha Kannan, Gregory Fontenot, Michael S. Nakazawa, Monzy Thomas, Khalida Wani, Clifford Stephan, Phyu P. Aung, Lawrence N. Kwong, Janos Roszik, Rehan Akbani, Andrew Kossenkov, Vito W. Rebecca, Ryan J. Sullivan, Meenhard Herlyn, Michael A. Davies

**Affiliations:** 1 Department of Melanoma Medical Oncology, University of Texas M.D. Anderson Cancer Center, Houston, TX USA; 2 Dept of Translational Molecular Pathology, University of Texas M.D. Anderson Cancer Center, Houston, TX USA; 3 Department of Medicine, Baylor College of Medicine, Houston, TX USA; 4 University of Texas Medical Branch, Galveston, TX USA; 5 Dept of Bioinformatics and Computational Biology, University of Texas M.D. Anderson Cancer Center, Houston, TX USA; 6 Ellen and Ronald Caplan Cancer Center, Wistar Institute, Philadelphia, PA USA; 7 Institute of Biosciences and Technology, Texas A&M University, Houston, TX USA; 8 Dept of Sarcoma Medical Oncology, University of Texas M.D. Anderson Cancer Center, Houston, TX USA; 9 Dept of Anatomic Pathology, University of Texas M.D. Anderson Cancer Center, Houston, TX USA; 10 Johns Hopkins Bloomberg School of Public Health, Baltimore, MD USA; 11 Cutaneous Medical Oncology Program, Mass General Brigham Cancer Institute and Harvard Medical School, Boston, MA USA; 12 Dept of Genomic Medicine, University of Texas M.D. Anderson Cancer Center, Houston, TX USA

**Keywords:** Cancer, Biomarkers

## Abstract

There are currently no effective targeted therapies for BRAF-mutant metastatic melanoma patients with acquired resistance to approved BRAF and MEK inhibitors (BRAFi and MEKi), and very few ongoing clinical trials. Anti-apoptotic BCL2 family proteins promote de novo resistance to several therapies, including single-agent BRAFi in BRAF-mutant melanomas. In this study, in vivo testing of a large collection of patient-derived xenograft (PDX) models from melanoma patients with acquired resistance to BRAFi or BRAFi+MEKi shows that combining BCL2 inhibitors (BCL2i; navitoclax or venetoclax) with BRAFi+MEKi induces tumor regressions in a subset of these PDXs. High basal BCL2 predicts response whereas high basal MCL1 predicts resistance to this strategy. MCL1 overexpression studies functionally validate its role in resistance. Further, combining BRAFi+MEKi with an MCL1 inhibitor (MCL1i) counteracts resistance and interestingly decreases MCL1i-associated markers of cardiotoxicity. Together these studies identify potential personalized strategies to improve outcomes in this challenging patient population.

## Abstract (web summary)

There is a critical unmet need to identify treatments that overcome acquired resistance to approved BRAF + MEK inhibitor combinations in BRAF-mutant melanomas. Here, the authors show that targeting BCL2 family protein members decreases resistance in a subset of BRAF-mutant preclinical melanoma models.

## Introduction

Melanoma is the most aggressive form of skin cancer. More than 75% of cutaneous melanomas harbor driver mutations that activate the RAS-RAF-MEK-ERK MAPK signaling pathway. Most commonly (~50% of patients) this is due to the presence of a missense mutation in the *BRAF* gene that results in a valine → glutamine substitution which markedly increases kinase activity of the resulting mutant BRAF^V600E^ protein, leading to constitutive activation of the MAPK pathway and promotion of melanoma growth^1,2^. This finding has been translated into clinically actionable drugs which specifically target BRAF^V600^ mutant melanomas. For example, the combination of Dabrafenib, a BRAF^V600^ inhibitor (BRAFi), with Trametinib, a MEK inhibitor (MEKi), is a standard of care therapy for metastatic melanomas with BRAF^V600^ mutation^3–5^. Although BRAFi + MEKi combination therapy achieves initial clinical responses in the overwhelming majority of patients, ~80% of melanoma patients develop acquired resistance to these agents within 2 years^4^. Further, only ~40% of metastatic melanomas achieve durable responses to immune checkpoint inhibitors- and the efficacy of immunotherapies is decreased in patients that have already progressed on BRAFi + MEKi^6–8^. Currently, there are no effective targeted therapy strategies for BRAF-mutant melanoma patients with acquired resistance to BRAFi + MEKi. Thus, there remains a significant unmet clinical need to identify strategies that overcome resistance to clinically approved therapies in BRAF^V600^-mutant melanoma patients.

Constitutively activated MAPK pathway in BRAF^V600^-mutant melanomas downregulates pro-apoptosis BCL2 family proteins, BIM and BAD, to promote melanoma cell survival^9^. Inhibition of the MAPK pathway upregulates these pro-apoptotic proteins, which bind to and inactivate the anti-apoptotic BCL2 family proteins, BCL2, BCL-xL and MCL1. This, in turn, promotes melanoma cell apoptosis and tumor growth inhibition in early stages of treatment^10,11^. However, the majority of melanoma patients eventually develop resistance to BRAFi. This coincides with high basal expression or adaptive upregulation of anti-apoptotic BCL2 family proteins, which promote resistance^12,13^. These findings support the rationale to test BCL2 targeting as a combination strategy with BRAFi for BRAF^V600E^ mutant melanoma patients. However, as BRAFi+MEKi combination is the current standard of care, it is unknown if BCL2 upregulation may also play a role in resistance to this combination therapy, and whether a triple combination of BRAFi+MEKi+BCL2i is warranted to overcome acquired resistance to BRAFi+MEKi therapy.

The BH3-mimetic Venetoclax (ABT-199) specifically inhibits BCL2 and is approved for treatment of hematological cancers, while Navitoclax (ABT-263) inhibits BCL2 and BCL-xL and is an experimental agent in clinical trials for hematological and solid cancers^14–16^. Clinical development of Navitoclax has been hindered by dose-limiting thrombocytopenia due to BCL-xL inhibition. However, the anti-tumor efficacy of BCL2-only inhibitors like Venetoclax is diminished by treatment-induced upregulation of BCL-xL, emphasizing the need to also inhibit BCL-xL^17^. Pre-clinical studies demonstrated that either inhibitor combined with targeted therapies can augment anti-tumor activity against solid cancers including melanomas, leading to clinical trials^18,19^. Previous BCL2i+BRAFi combination studies in melanoma were performed using cell lines or tumor xenografts without molecular stratification and failed to identify molecular predictors of sensitivity^13,20–22^. Identification of predictive markers using patient-relevant models could facilitate the selection of melanoma patients who would most benefit from these treatments. In the current study, we performed combination treatments of Navitoclax or Venetoclax with Dabrafenib + Trametinib in a large collection of clinically annotated and molecularly characterized BRAFi/MEKi-resistant melanoma PDXs. These PDXs were generated from tumor biopsies of melanoma patients who developed progressive disease while on FDA-approved targeted therapy regimens^23^. Compared to traditional pre-clinical tumor models, these low passage in vivo propagated PDXs better replicate therapeutic responses attributable to individual patients^24^. Our results identified molecular determinants of sensitivity and resistance to this triple combination treatment in BRAF-mutant melanomas with acquired resistance to BRAFi + MEKi, as well as additional potential strategies to build upon the activity of that regimen.

## Results

### BCL2 protein is heterogeneously expressed in melanomas and is upregulated in response to MAPK inhibitor treatments in patient tumors

**Fig. 1:**
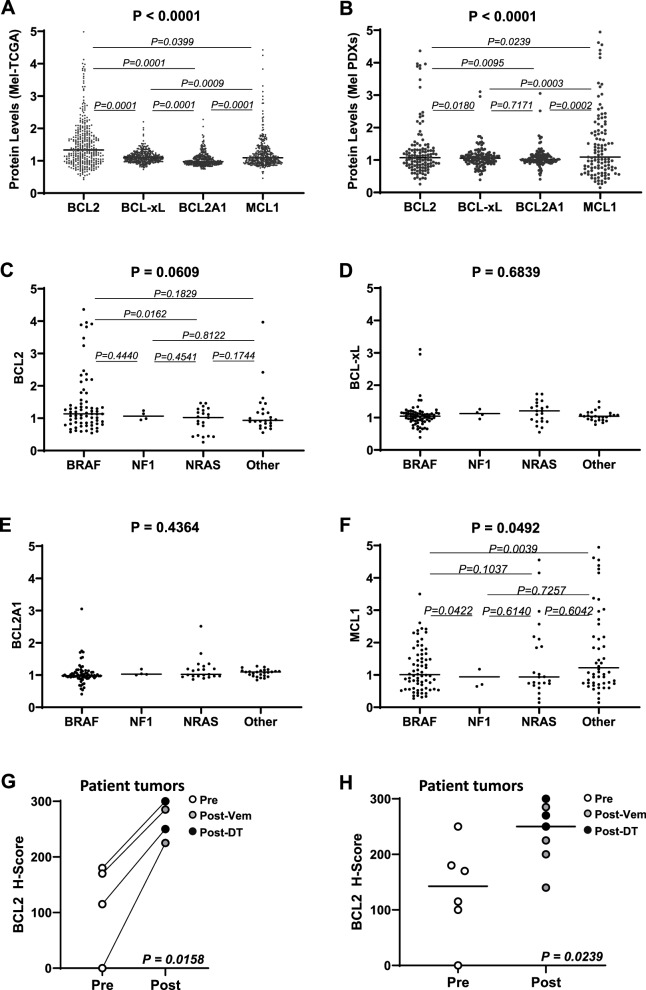
BCL2 family protein expression in melanoma. Scatter plots showing expression of BCL2, BCL-xL, BCL2A1 and MCL1 proteins in the TCGA melanoma patient tumor samples (*n* = 355) (**A**) and melanoma PDX tumor samples (*n* = 119) (**B**) analyzed from RPPA data. Scatter plots showing expression of BCL2 (**C**), BCL-xL (**D**), BCL2A1 (**E**) and MCL1 (**F**) in melanoma PDX tumors with the three main driver mutations BRAF, NF1, NRAS or Other. For figures *A* through *F*, significance was assessed using one-way ANOVA between all comparison groups, and post-hoc two-sided *t*-tests between individual groups. Horizontal lines identify mean of each scatter and significant differences between groups are indicated by *P*-values if *P* < *0.05* between any individual groups. BCL2 protein levels are represented as H-scores, in patient-matched (**G**) and unmatched (**H**) melanoma tumors pre- and post-treatment with Vemurafenib (Vem) or Dabrafenib+Trametinib (DT). Data recorded from five random 1 mm^2^ areas in each patient tumor and significance determined by two-sided t tests.

Reverse Phase Protein Array (RPPA) analysis of up to 32 different cancer types in The Cancer Genome Atlas (TCGA) database showed that melanomas have amongst the highest expression of the anti-apoptotic BCL2 and BCL-xL (Fig. S1A and S1B) but lower levels of BCL2A1 and MCL1 (Fig. S1C and S1D) proteins^2^. Melanomas exhibit significant heterogeneity of expression of all four proteins (one-way ANOVA *P* < 0.0001), with BCL2 and MCL1 showing higher average relative expression among the TCGA samples compared to BCL-xL and BCL2A1 (BCL2 = 1.51, MCL1 = 1.38, BCL-xL=1.11, BCL2A1 = 1.03) (Fig. 1A). RPPA analysis of 173 molecularly characterized melanoma PDX models^23^ showed similar heterogeneity of expression of these proteins (one-way ANOVA *P* < 0.0001), and higher average relative expression of BCL2 and MCL1 (BCL2 = 1.27, MCL1 = 1.67, BCL-xL=1.08, BCL2A1 = 1.06) (Fig. 1B). These results show that PDX models represent the molecular diversity of anti-apoptotic BCL2 family proteins found in melanoma patient tumors.

When the PDXs were stratified by the presence of MAPK pathway activating mutations in *BRAF*, *NF1*, *NRAS* or ‘Other’ (triple wildtype) genes, the levels of BCL2, BCL-xL and BCL2A1 were similar among PDXs of these mutation types, while MCL1 levels were higher in triple wildtype (one-way ANOVA *P* < 0.0492) (Fig. 1C–F). MCL1 (one-way ANOVA *P* < 0.0451) and BCL2 (one-way ANOVA *P* = 0.0001) levels were also associated with MAPK pathway mutation status in the melanoma TCGA cohort (Fig S1E–S1H). Earlier studies implicated MAPK inhibitor (MAPKi)−induced differential alterations in anti-apoptotic BCL2 family proteins with de novo resistance to BRAF1/MEKi treatments^13,20–22^. To specifically probe the role of BCL2, we evaluated pre- and post-treatment BCL2 levels in tumors biopsied from melanoma patients whose disease had progressed after treatment with the BRAFi, Vemurafenib (Vem) or with the BRAFi+MEKi, Dabrafenib+Trametinib (DT) (Fig S1I). In four patient-matched tumors, IHC staining (quantified as H-scores) revealed >1-fold increase of BCL2 levels in all post-treatment versus pre-treatment tumors (*P* = 0.015) (Fig. 1G). Including additional unmatched pre- and post-treatment clinical samples (Fig. S1I) also demonstrated significant increase of BCL2 (*P* = 0.024) following Vem or DT treatments (Fig. 1H). These results, together with previous studies implicating MAPKi−induced BCL2 proteins with resistance^13,20,22^, suggest that BCL2 upregulation may confer resistance to BRAFi and BRAFi+MEKi, and support the rationale to inhibit BCL2 along with BRAF and MEK to prevent or overcome resistance.

### Anti-tumor activity of BRAFi/MEKi combination with BCL2i in melanoma PDXs

**Fig. 2:**
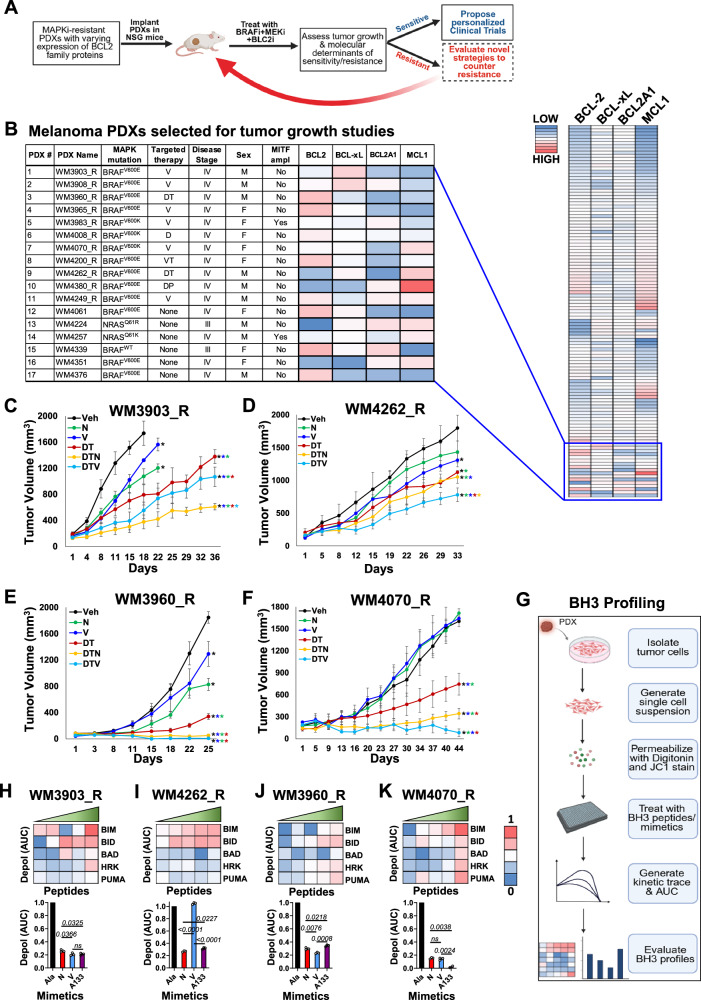
PDX tumor selection and evaluation of BCL2 inhibitor combination with BRAF and MEK inhibitors. **A** Workflow of PDX selection for tumor growth studies, drug treatments and assessment of molecular features of treatment response; Created in BioRender. Loftin, K. (2026) https://BioRender.com/8we1hak. **B** Heatmap on the right shows expression of anti-apoptosis BCL2 family proteins in molecularly and clinically annotated melanoma PDXs, and table on the left shows PDXs selected for in vivo testing of BCL2i combination with BRAFi/MEKi. PDX names ending in “_R” are from patients with progressive disease who were on the indicated targeted therapy at the time of patient tumor biopsy. In column ‘Targeted Therapy’, V is Vemurafenib, D is Dabrafenib, T is Trametinib, *P* is Pembrolizumab. When together, they indicate combination treatments. **C**−**F** Tumor growth of PDXs in mice treated daily with vehicle (Veh, black), 75 mg/kg Navitoclax (*N*, green), 75 mg/kg Venetoclax (V, dark blue), 30 mg/kg Dabrafenib + 1 mg/kg Trametinib (DT, red) or their combinations, DTN (yellow) or DTV (light blue). Data represents mean of ≥6 mice +/− SEM. Significance was determined by two-way ANOVA and Tukey’s multiple comparisons tests; significant difference (*P* < 0.05) between a growth curve versus another growth curve is indicated by asterisks of the same color as the other growth curve, and numerical *P*-values shown in Fig S1J. **G** Schematic of BH3 profiling assay performed using JC1 staining of PDX-derived cells; Created in BioRender. Loftin, K. (2026) https://BioRender.com/z5nkkm0. **H**−**K** Heatmaps (top) and bar graphs (bottom) showing mitochondrial membrane depolarizations induced by BH3 peptides (shown beside heatmaps) and BH3 mimetics (shown under x-axis of bar graphs) for each of the four PDXs. Green triangles over the heatmaps indicate increasing concentration of BH3 peptides (1 µM, 10 µM, 50 µM, 100 µM, and 500 µM) and the heatmap scale indicates depolarization range of 0–1, with 0 corresponding with negative control PUMA2A in blue, and 1 corresponding with positive control Ala (25 µM Alamethicin) in red. In the bar graphs, BH3 Mimetics were 300 nM of N, V and A133 (BCL-xLi, A1331852). Ala, positive control and DMSO, negative control. Data represents mean of biological triplicates +/− SD and significant differences were determined by two-sided t-tests.

Given the observed heterogeneity in the expression of anti-apoptosis BCL2 family proteins, we performed tumor growth inhibition studies in PDX models with representative heterogenous expression of these proteins. Using the strategy outlined in Fig. 2A, we selected PDXs with varying levels of anti-apoptotic BCL2 family proteins (table in Fig. 2B) from a collection of 117 melanoma PDX models (heatmap in Fig. 2B). Among the selected were PDXs (*n* = 11) established from BRAF-mutant melanoma patients with progressive disease while on FDA-approved targeted therapies (BRAFi or BRAFi+MEKi) and which were maintained on BRAFi/MEKi treatments in mice to sustain this key phenotype. Also included were representative PDXs from targeted therapy treatment−naïve BRAF-mutant (*n* = 3) or BRAF-wildtype (*n* = 3) melanoma patients who subsequently did not respond to BRAFi or MEKi treatments. Two of the selected PDXs had MITF amplification; MITF-induced BCL2A1 activation was previously implicated in resistance to BCL2i^25^.

We then evaluated the anti-tumor activity of BCL2i in combination with BRAFi/MEKi in these PDXs grown as subcutaneous tumors in NSG mice. Mice with BRAF^V600^-mutant PDX tumors were treated with vehicle control; single agents Navitoclax (N; 75 mg/kg) or Venetoclax (V; 75 mg/kg); Dabrafenib + Trametinib (DT; 30 mg/kg D + 1 mg/kg T); or triple combinations (DTN or DTV). In mice with BRAF wildtype PDX tumors, DT was replaced with T only and DTN was replaced with TN. Treatment-induced changes in tumor growth were assessed after three weeks or more. We defined treatment responses by following the new NCI-PDXnet consensus recommendations that tumor growth stabilization (stasis) or tumor regression are the benchmarks of clinically meaningful treatment responses, as opposed to statistically significant but small tumor growth inhibition^26^. The results showed that N or V as single agents had no effect on tumor growth or they induced statistically significant growth inhibition that was quantitatively small without stasis or regression (four PDXs in Fig. 2C–F; all others in Figs. S1A–S2M). Treatment with DT or T induced either no effect or statistically significant growth inhibition that was small to intermediate in all PDXs except in one which underwent tumor stasis (Fig. S2J), suggesting that all but one PDX are completely or moderately resistant to standard of care MAPK inhibition. However, DTN and DTV treatments were significantly more effective, with multiple BRAFi/MEKi−resistant PDXs undergoing stasis or regression (Fig. 2C–F; Figs. S2A–S2M).

To understand treatment responses to N and V as a function of their molecular activities, we performed a BH3 profiling assay (Fig. 2G) which uses BH3 peptides or mimetics treatments to identify activities of pro- and anti-apoptosis BH3 proteins that ultimately determine if a specific tumor cell is primed towards cell death (Fig. S3A), and measured as mitochondrial membrane depolarization. The binding affinities of BCL2 family proteins with apoptosis activators (BIM and BID), sensitizers (BAD, HRK, PUMA), and BH3 mimetics (N, V, A1331852) used in this assay are shown in Fig. S3B and S3C. Results of BH3 profiling assay on the four PDXs from Fig. 2C–F are shown in Fig. 2H–K (heatmaps represent depolarizations induced by BH3 peptide treatments; bar graphs represent depolarizations induced by BH3 mimetic treatments. WM3903_R and WM4262_R, which underwent significant growth inhibition but not stasis or regression with DTN and DTV treatments, showed high ( > 0.5) depolarizations with increasing concentrations of BIM and BID, and low ( < 0.5) depolarizations by PUMA. WM3960_R and WM4070_R, which underwent tumor regression with DTN and DTV treatments, showed higher depolarizations by PUMA. Activation of PUMA by BCL2 family inhibitors sensitizes tumor cells to combination treatments^27^ leading to tumor regressions in these PDXs. With respect to differences in response to BCL2 and BCL-xL inhibition, one of the four PDXs (WM4262_R) exhibited lower depolarization with the BCL-xL targeting peptide, HRK (Fig. 2I). BH3 mimetics profiling also showed lower depolarization with N and A1331852 compared to V suggesting lower responsiveness to BCL-xL inhibition compared to BCL-2 inhibition. This was also reflected in the increased tumor growth inhibition by DTV versus DTN in this PDX model (Fig. 2D).

### Molecular features of response to BRAFi + MEKi + BCL2i treatments

**Fig. 3:**
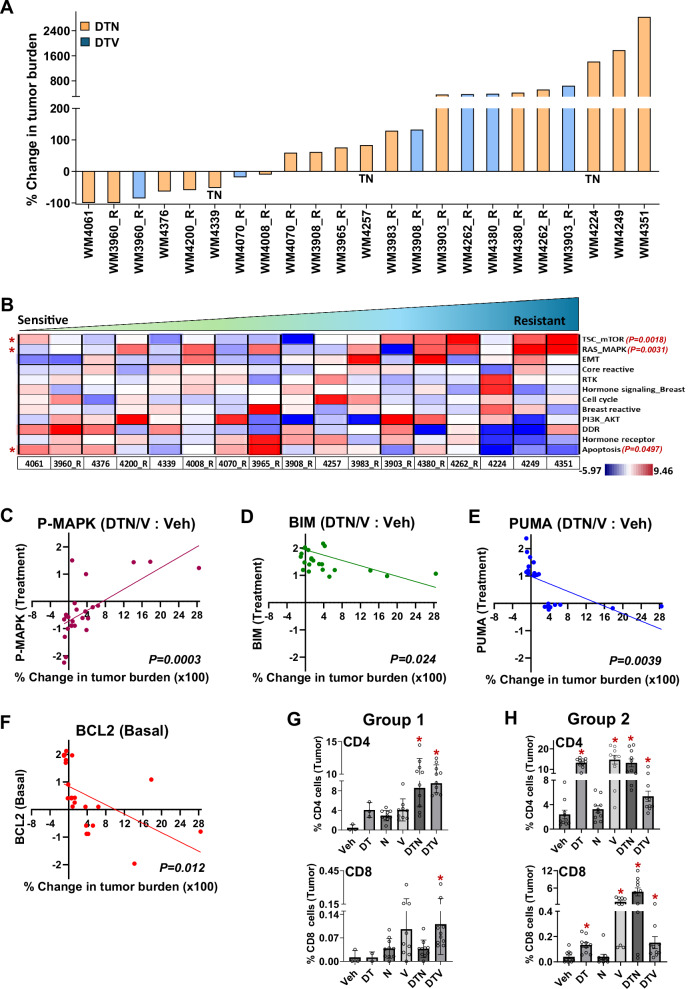
Treatment induced changes in PDX tumor burdens and molecular associations. **A** Column graph showing lowest to highest (left to right) percentage changes in PDX tumor burdens from the start to end or maximum ~30 days of treatment. Yellow bars represent DTN and TN treatments, with TN treatments indicated under the respective columns, and blue bars represent DTV treatments. Treatments were 75 mg/kg Navitoclax (N), 75 mg/kg Venetoclax (V), 30 mg/kg Dabrafenib (D), 1 mg/kg Trametinib (T) as daily doses of single or combination treatments. **B** Supervised Pearson correlation hierarchical clustering heatmap of the basal levels of 12 protein signaling networks in PDXs with lowest (left) to highest (right) tumor burdens. PDX names are shown at the bottom of the heatmap. Significantly correlating signaling networks with tumor burden determined by two-sided t-tests are indicated with asterisks (left) and *P*-values (right). Linear regression analysis of the association of DTN/V-treatment induced P-MAPK (**C**), BIM (**D**) and PUMA (**E**) proteins with DTN/V treatment-altered changes in PDX tumor burdens. Significance determined by two-way best fit analysis, and *P* values indicated at the bottom right corner of each graph. **F** Linear regression analysis of the association of basal BCL2 protein with percent changes in PDX tumor burdens following treatments with DTN/V. *P* value derived from two-way best-fit analysis indicated at the bottom right corner. **G**, **H** IHC analysis of tumor-infiltrated CD4 and CD8 T cells shown as percent positive staining cells in WM4380_R PDX tumors grown in two groups of CD34+ humanized mice, treated daily with the indicated therapeutic agents. Data represents mean of ten random 1 mm^2^ tumor measurements per treatment +/− SD. Significance determined by one-way ANOVA and Kruskal–Wallis multiple comparisons testing and *P* < 0.05 indicated by asterisks. *P*-values: (**G**) CD4: Veh vs DTN, *0.0024*; DTV, *0.0006*; CD8: Veh vs DTV, *0.0091*; (**H**) CD4: Veh vs DT, *<0.0001*; V, *<0.0001*; DTN, *<0.0001*; CD8: Veh vs DT, *0.059*; V, *0.0001;* DTN, *<0001; DTV, 0.0090*.

To identify molecular features of response or resistance from the above PDX tumor growth inhibition study, we first determined treatment-induced changes in tumor burdens at ~30 days, or less for shorter treatments, versus at the start of treatments. Neither single agent N or V induced tumor stasis or tumor regressions in any of the PDXs (Fig. S3E). DT also did not induce stasis or regression, although treatment with T induced stasis of one PDX (Fig. S3F). However, DTN and DTV induced tumor regressions in seven PDXs, four of which were established from patients progressing on FDA-approved targeted therapies (Fig. 3A). Sex-disaggregated analysis did not show any significant differences in tumor growth inhibition of PDXs from male or female patients (Fig. S3D for DTN, Fig. S3E and S3F insets for N/V and DT). We then evaluated the association of change in tumor burden with the basal activation status of 12 protein signaling pathways in tumor protein lysates by RPPA analysis as previously described^28^. Pearson’s correlation analysis of the activation status of these pathways showed a significant upregulation of apoptosis pathway with sensitivity to DTN and DTV, and a significant upregulation of TSC-mTOR and RAS-MAPK pathways with resistance (Fig. 3B).

Gene Set Enrichment Analysis (GSEA) of RNA-seq data from eight PDXs treated with DTN and six with DTV showed highly significant downregulation (FDR < 0.0001) of the same cell cycle regulatory networks by these treatments in the sensitive versus resistant PDXs (Fig. S3G and S3H). DTV treatment downregulated additional transcriptional networks (0.0001 < FDR < 0.01) in the sensitive PDXs, which is interesting given that V is a selective BCL2i versus N. These molecular differences could be evaluated in clinical studies in the future to assess the significance of N versus V for patient responses.

Treatment-induced alterations in protein levels were determined by RPPA analysis of tumor lysates around seven days of treatment. Linear regression analysis showed that reduction of tumor burdens by DTN and DTV compared to vehicle (DTN/V: Veh) correlated significantly with treatment-induced downregulation of Phospho-MAPK (R^2^ = 0.4472, *P* = 0.0003; Fig. 3C) and upregulation of BIM (R^2^ = 0.3025, *P* = 0.024; Fig. 3D), which are known markers of MAPK and BCL2 inhibition. Reduction of tumor burden also significantly correlated with treatment-induced upregulation of the apoptosis sensitizer PUMA (R^2^ = 0.3466, 0.0039; Fig. 3E). BH3 profiling of cells from eight of these PDXs showed that PUMA-induced depolarization correlated significantly with DTN/V treatment-induced PUMA protein levels (R^2^ = 0.5605, *P* = 0.0326, Fig S3I), affirming its functional role as observed in the BH3 profiling result in Fig. 2H–K. Interestingly, reduction of tumor burden by DT only treatment showed similar but non-significant correlations with the two markers (Phospho-MAPK, *P* = 0.2282; BIM *P* = 0.1889; Fig. S3J and S3K). Tumor burden reduction by N or V only treatments showed a significant but weaker correlation with BIM upregulation (R^2^ = 2068, *P* = 0.0224; Fig S3L) compared to DTN/V treatments. Together, these results suggest that increased target inhibition by the combination of BRAFi/MEKi with BCL2i/BCL-xLi increases tumor growth inhibition.

DTN/V treatment-induced reduction of tumor burden also correlated significantly with basal BCL2 protein levels (R^2^ = 0.2873, *P* = 0.0123, Fig. 3F) and non-significantly with basal BCL-xL levels (R^2^ = 0.1559, *P* = 0.0765, Fig. S3M) in the PDXs. However, BH3 profiling of cells from eight of these PDXs showed a non-significant association of DTN-induced depolarization with BCL2 and no association with BCL-xLi protein levels (BCL2 R^2^ = 0.3738, *P* = 0.1073, Fig S3N; BCL-xL R^2^ = 0.1655, *P* = 0.6982, Fig S3O). Additionally, as V inhibits BCL2 with a 100-fold higher potency than N but inhibits BCL-xL with a 100-fold lower potency than N (Fig. S3C)^29,30^, we tested whether basal BCL2 and BCL-xL levels in the PDXs show differential responses to DTV and DTN. Linear regression analysis showed that reduction of tumor burdens by DTN treatment correlated significantly with high BCL2, but non-significantly with high BCL-xL (BCL2 R^2^ = 3074, *P* = 0.0209; BCL-xL R^2^ = 0.1854, *P* = 0.0845; Fig S3P). Reduction of tumor burdens by DTV treatment showed a weak non-significant correlation with high BCL2 and no meaningful association with BCL-xL (BCL2 R^2^ = 0.4787, *P* = 0.1278; BCL-xL R^2^ = 0.1304, *P* = 0.4818, Fig. S3Q). Taken together, these results suggest that the anti-tumor activity of DTN and DTV combination treatments may have a higher dependency on basal BCL2 levels.

As MAPK inhibition increases immune T cell infiltration of tumors^31^ as was also shown for BCL2 inhibition^32–35^, we evaluated the effect of the combination treatments on CD4+ and CD8 + T cell infiltration into a *BRAF*^*V600E*^*−*mutant PDX (WM4380_R) grown in NSG-SGM3 humanized mice. IHC analysis of tumors from two separate groups of mice showed heterogenous effects, but none of the treatments inhibited CD4+ or CD8 + T cell infiltration into tumors (Fig. 3G, H). Compared to vehicle treatments, DT, N and V mostly induced non-significant increases, although in group 2 mice, DT induced a significant increase of CD4+ cell infiltration and V induced a significant increase of CD8+ infiltration (Fig. 3H). V, DTV and DTN induced significant or non-significant increases of CD4+ and CD8+ cells in both groups. Together, the results suggest that the single agent and combination treatments do not decrease tumor infiltration by these immune cell types (Fig. 3G, H; spleen control data in Fig. S3R, S3S).

### Long-term effects of treatments in DTN responding tumors

**Fig. 4:**
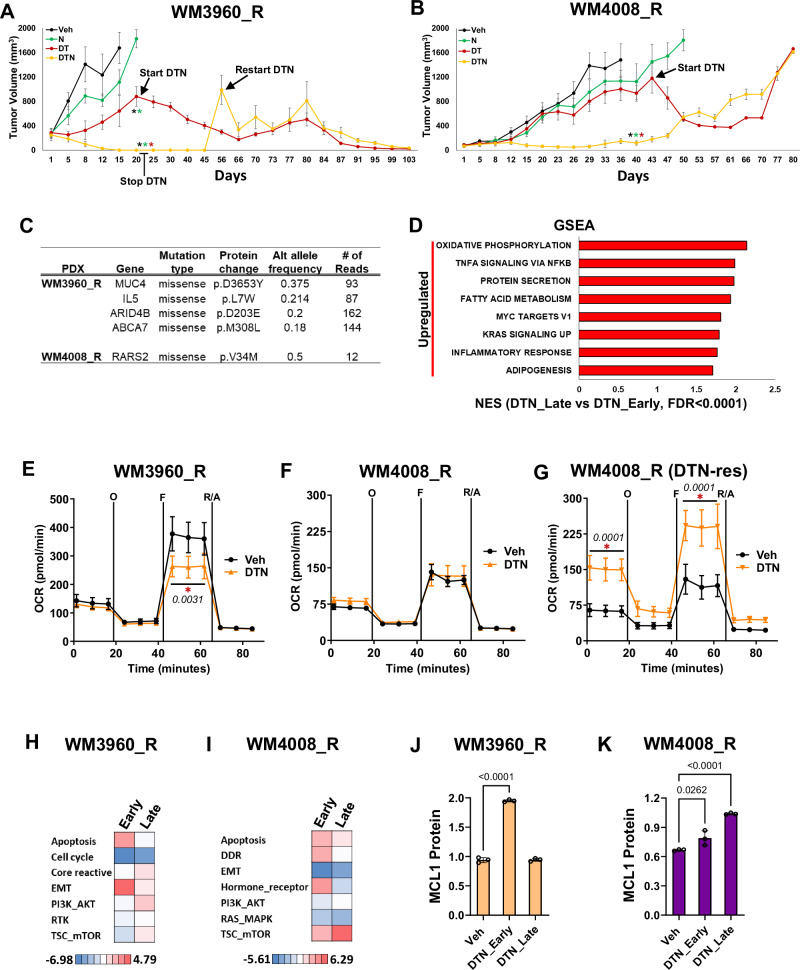
Molecular features of resistance to BRAFi + MEKi + BCL2i treatment. Long-term tumor growth of WM3960_R (**A**) and WM4008_R (**B**) treated with vehicle (Veh, black), Navitoclax (N, green), Dabrafenib+Trametinib (DT, red) or their combination, DTN (yellow). Change of DT treatments to DTN is indicated by arrows on the DT (red) growth curves. Data represents mean of ≥6 mice +/− SEM. Significance was determined by two-way ANOVA and Tukey’s multiple comparisons tests; significant difference (*P* < 0.05) of any growth curve versus others indicated by asterisks of the same color as the other growth curves. *P*-values of 20 days of treatment for WM3960_R and 40 days of treatment for WM4008_R are shown in the tables in Fig S4F-G. **C** Table showing new treatment induced mutations in late-harvest PDXs after 80 days of treatment with DTN. **D** GSEA analysis showing the normalized expression scores (NES) of most significantly altered (*FDR* = *0*) transcriptional networks in RNA of late compared to early harvest tumors extracted from DTN treated mice. Seahorse bioenergetics stress tests performed in cells from WM3960_R (**E**), WM4008_R (**F**) and late harvest WM4008_R (**G**) tumors from mice treated with vehicle or DTN. Oligomycin, ‘O’, FCCP, ‘F’, and Rotenone+Antimycin, ‘R/A’, were added to cells at the indicated times. Data is mean of five independently treated replicates +/− SD; Significance determined by two-sided *t* tests for the central measurement of each group and shown as *P*-values. Data represents reproducible results from two separate experiments. Differential activation status of protein signaling networks in the late versus early harvest WM3960_R (**H**) and WM4008_R (**I**) tumor lysates from RPPA analysis. MCL1 protein levels in Vehicle versus early and late harvest DTN treated tumors from WM3960_R (**J**) and WM4008_R (**K**). Data is mean of three independent replicates +/− SD; *P-*values are from two-sided *t*-tests.

Based on the above short-term ( ~ 30 day) tumor growth inhibition results, we evaluated long-term ( > 75 days) effects in two DTN-sensitive PDXs, WM3960_R and WM4008_R (Fig. 2E and S2G). DTN treatment of tumor bearing mice induced complete regressions of WM3960_R within 15 days (Fig. 4A). Withdrawal of treatment after 20 days resulted in tumor re-growth ~25 days post-withdrawal, which was reversed upon re-treatment with DTN in seven of eight mice (Fig. 4A). One mouse showed increased growth compared to others at 80 days of treatment and was harvested to evaluate molecular markers of potential acquired resistance. In the same experiment, mice undergoing treatment with DT showed an initial decrease of tumor growth rate compared to vehicle control or N, which however was not sustained beyond the first week of treatment. Changing treatment from DT to DTN at day 20 resulted in tumor regression. Eventually, neither group undergoing DTN treatments had tumor regrowth after 100 days (Fig. 4A), indicating long-term efficacy of DTN in this PDX model. Interestingly, in the WM4008_R model, DTN treatment of tumor-bearing mice achieved incomplete tumor regressions within 20 days of DTN treatment, but tumor re-growth occurred after ~40 days while still on treatment, consistent with the development of acquired resistance (Fig. 4B). The DT treatment mice exhibited significant inhibition of tumor growth, but not tumor regression. Changing treatment from DT to DTN at day 43 resulted in tumor regression, which however was not sustained beyond ~30 days of initiating DTN treatment in these mice (Fig. 4B). These results suggest that early and complete regression with DTN may result in long-term tumor eradication in some melanomas (i.e., WM3960_R), whereas incomplete regression in others (i.e., WM4008_R) may lead to acquired resistance and resumption of tumor growth.

The molecular features of tumors harvested at seven days (Early harvest) and 80 days (Late harvest) of DTN treatment were evaluated to identify potential resistance-causing alterations. Whole genome sequencing (WGS) analysis of DNA revealed protein-altering missense mutations in four genes from the single WM3960_R late harvest tumor, none of which had alternate allele frequencies >40% (Fig. 4C). Similarly, a late-harvest WM4008_R tumor had one missense mutation with 50% alternate allele frequency, but in only 12 reads (Fig. 4C). These results in the late harvest tumors from both PDX models suggest the existence of clonally heterogenous mutations, but the low allelic frequency and read numbers suggest that neither alteration likely explains the observed resistance.

GSEA of RNA-seq data identified oxidative phosphorylation as the most significantly upregulated transcriptional network in the Late versus Early harvest tumors of the PDXs (Fig. 4D, Fig S4A). Measurement of oxygen consumption rate (OCR), a surrogate for oxidative phosphorylation, in cells extracted from early harvest tumors showed that DTN treatment did not alter basal OCR in WM3960_R and WM4008_R, while it inhibited FCCP (F)-induced maximal OCR in WM3960_R only (Fig. 4E, F). We could not generate DTN resistance in WM3960_R beyond the one tumor observed in the tumor growth experiment and hence could not evaluate late harvest OCR in this PDX. However, DTN resistance in WM4008_R was replicable, and cells from this late harvest DTN resistant tumor showed significantly higher basal and maximal OCR compared to vehicle (Fig. 4G). Pathway analysis of RPPA quantified proteins identified seven unique protein signaling pathways in each PDX with >1-fold difference between Early harvest and Late harvest tumors (Fig. 4H, I). Downregulation of apoptosis and upregulation of TSC/mTOR pathways were the similar alterations in Late harvest resistant tumors of both PDXs (Fig. 4H, I). Taken together, these molecular findings suggest non-genetic modes of DTN treatment-acquired resistance. Notably, among the several proteins that showed differential alterations in the late harvest and early harvest tumors (Fig. S4B–E), MCL1 was upregulated in the early harvest tumors of both PDXs and in DTN-resistant WM4008_R late harvest tumors, but not in the WM3960_R late harvest tumor (Fig. 4J, K).

### MCL1 is a predictor of therapeutic resistance to BRAFi + MEKi + BCL2i treatment

**Fig. 5:**
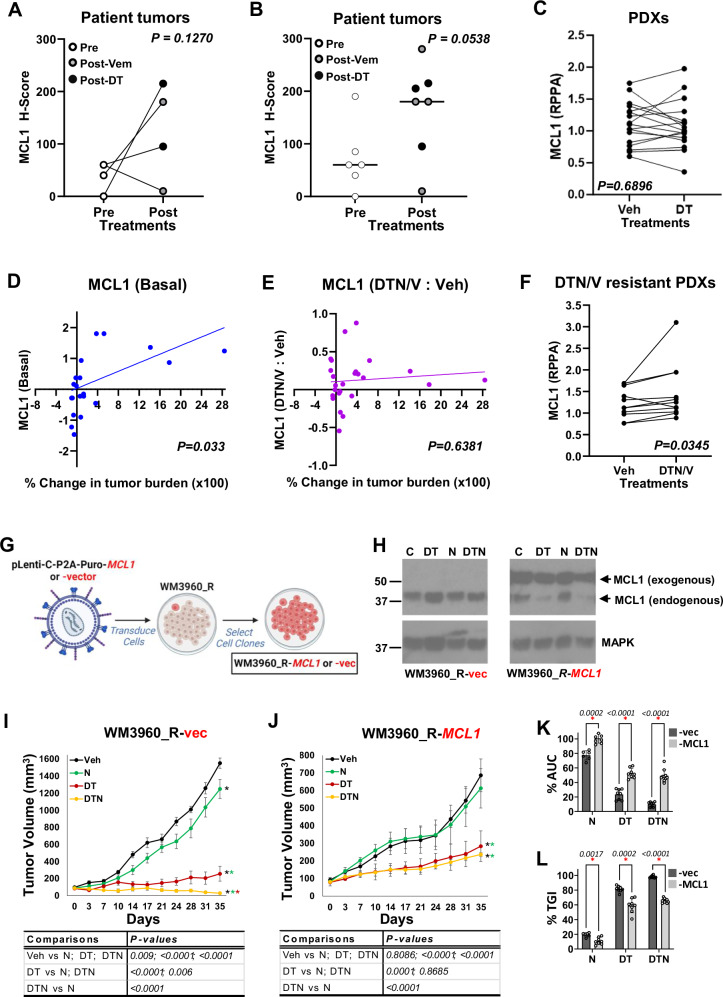
Role of MCL1 in treatment responses. MCL1 protein levels in patient-matched (**A**) and unmatched (**B**) melanoma tumors pre- and post-treatment with Vemurafenib (Vem) or Dabrafenib+Trametinib (DT), determined by H-score analysis of IHC data. Data is mean of five random 1 mm^2^ areas in each patient tumor and significance (*P* < *0.05*) determined by two-sided *t*-tests. **C** MCL1 protein levels in PDX-matched Veh and DT treated tumors of 17 PDXs. Data is mean of biological triplicates and significance determined using two-sided t-tests. Linear regression analysis of association of basal MCL1 (**D**) and DTN/V treatment-altered MCL1 (**E**) protein levels with changes in tumor burden in the 17 PDXs. Significance (*P* < 0.05) by best-fit analysis. **F** MCL1 protein levels in Veh and DTN/V treated tumors of DTN/V-resistant PDXs. Data is mean of biological triplicates and significance determined using two-sided t-tests. **G** Generation of MCL1-overexpressing clones or its vector control (WM3960_R-*MCL1* or -*vec*) in WM3960_R PDX-derived cells; Created in BioRender. Loftin, K (2026) https://BioRender.com/cbh7s6w. **H** Western blotting of exogenous and endogenous MCL1 protein in cells after treatment with the indicated drugs. MAPK (ERK2) protein was used as loading control and was detected on the same membranes as MCL1 after stripping the membranes. Molecular weight markers in kDa are indicated on the left of each blot. Data represents reproducible results from two separate experiments. Tumor growth of WM3960_R-*vec* (**I**) and WM3960_R-*MCL1*(**J**) in mice treated daily with vehicle (Veh, black), 75 mg/kg Navitoclax (N, green), 30 mg/kg Dabrafenib+1 mg/kg Trametinib (DT, red) or their combination, DTN (yellow). Data represents mean of ≥6 mice +/− SEM. Significance determined by two-way ANOVA and Tukey’s multiple comparisons tests; significant difference (*P* < 0.05) of any growth curve versus others is indicated by asterisks of the same color as the other growth curves and *P*-values shown in the below tables. **K** Bar graphs of Area Under the Curve (AUC) analysis of data from **I** and **J** showing percent change of tumor sizes in treatments versus vehicle. Significance determined by two-sided *t*-tests. **L** Tumor Growth Inhibition (TGI) by the treatments in **I** and **J** shown as percent changes versus vehicle. Significance determined by two-sided t-tests.

The above observation suggests that MCL1 expression could be an important predictor of clinical responses to DTN treatment. However, a quantifiable metric to assess MCL1 protein levels in clinical samples was lacking. For that, we validated an MCL1 antibody for IHC and analyzed 293 tumors from stage IV melanoma patients to develop an H-scoring metric for MCL1 by blinded scoring by two dermatopathologists (Fig. S5A). The results did not show a sex difference in basal tumor MCL1 levels (Fig. S5B) and showed that 76.4% tumors have zero to low MCL1 (H-score 0–75), 14.4% medium (H-score 76–150), and 8.9% high (H-score 151–300) MCL1 levels (Fig S5C). These results suggest that basal MCL1 over-expression is rare in melanomas, as was also observed in RPPA analysis of the TCGA samples (Fig. S1D). We then determined BRAFi/MEKi treatment effects on MCL1 levels using IHC H-scores in the cohort of tumors from melanoma patients (Fig. S1I) treated with DT or Vem and assessed for BCL2 (Fig. 1G, H). The results showed that MCL1 levels were upregulated >1-fold in only two of four patient-matched post-treatment tumors (Fig. 5A), unlike BCL2 which was upregulated >1-fold in all four post-treatment tumors (Fig. 1G). Analysis of patient-matched and unmatched tumors together showed a non-significant upregulation of MCL1 in DT/Vem post-treatment tumors (Fig. 5B), also unlike BCL2 which was upregulated significantly in all post-treatment tumors (Fig. 1H). Analysis of MCL1 levels in vehicle and DT treatment PDXs similarly showed that DT treatment increased MCL1 levels in only nine of 17 PDXs, validating the heterogenous effect of BRAFi/MEKi treatment on MCL1 expression in melanoma (Fig. 5C).

Basal MCL1 protein in the 17 melanoma PDXs significantly correlated with resistance to DTN and DTV (DTN/V) (*P* = 0.033, Fig. 5D). However, similar to DT treatments in patient tumors and PDXs, DTN/V treatment differentially altered MCL1 in the 17 PDXs (Fig. S5D) and did not show an overall association with change in tumor burden (*P* = 0.6381, Fig. 5E). However, DTN/V treatment-resistant PDXs (defined as tumor burden increase of >100% at ~30 days compared to start of treatment) showed a significant increase of MCL1 in treatment versus vehicle (*P* = 0.0345, Fig. 5F), but the sensitive PDXs did not show a significant change (*P* = 0.9171, Fig. S5E). Additionally, preliminary unpublished analysis of tumors from two DTN treatment-progressed melanoma patients in a clinical trial^36^ identified an increase of MCL1 protein in the resistant tumors compared to pre-treatment tumors (not shown). Together, our results indicate that basal MCL1 upregulation is rare in melanomas; BRAFi/MEKi treatments and their combination with BCL2i may upregulate MCL1 levels in some but not all treatment-resistant melanomas; and that treatment-induced upregulation of MCL1 is a potential mechanism of resistance to triple combination therapy with DTN/V.

### Enforced expression of MCL1 causes resistance to BRAFi + MEKi + BCL2i

To functionally validate the role of MCL1 upregulation in resistance to DTN/V, we overexpressed MCL1 in two PDX-derived cell lines: WM3960_R (*BRAF*^*V600E*^-mutant, low MCL1 expression and sensitive to DTN/V); and WM4257 (*BRAF*-wildtype, moderate MCL1 expression and moderately resistant to TN). The cell lines were transduced with lentiviral constructs expressing *MYC*-tagged human *MCL1* gene or tagged vector control (-vec), and expression-positive cell clones were isolated using puromycin selection (Fig. 5G, Fig. S6A). Western blotting analysis showed robust expression of exogenous MCL1 protein in the WM3960_R-*MCL1* and WM4257-*MCL1* cells (Fig. 5H, Fig. S6B). DT/N/V treatments induced varying changes in endogenous MCL1 protein levels in the -*MCL1* cells, but did not alter exogenous MCL1 levels, suggesting transcriptional regulation of endogenous MCL1 expression by these treatments (Fig. 5H, Fig. S6B). We then generated sub-cutaneous xenografts of the -vec and -*MCL1* cells in mice, and performed treatments with DT, N or DTN in WM3960_R and T, V or TV in WM4257 tumor-bearing mice, respectively. The *-MCL1* tumors had an overall slower growth rate compared to control -vec tumors, with WM3960_R-*MCL1* growing at half the rate as the control WM3960_R-vec (Fig. 5I, J), and WM4257-*MCL1* growing ~20% slower than control WM4257-vec (Figs. S6C and S6D). Compared to -vec tumors (Fig. 5I and Fig. S6C), -*MCL1* tumors (Fig. 5J and S6D) were significantly resistant to growth inhibition by single agent N or V or DTN/DTV combination treatments. Importantly, DTN induced tumor regressions in WM3960_R-vec, but not in WM3960_R-*MCL1*, where it induced only significant growth inhibition. Tumor burdens over the entire treatment periods were assessed using AUC analysis which revealed that DTN treatment induced a nine-fold decrease of tumor burden in the WM3960_R-vec tumors, but only a five-fold decrease in the WM3960_R-*MCL1* tumors, compared to vehicle treatments (Fig. 5K). Tumor growth inhibition (TGI) at the conclusion of treatments were assessed as percent-changes versus vehicle, which showed that DTN induced near complete regression of WM3960_R-vec tumors (98% versus vehicle), but not the *-MCL1* tumors (65% versus vehicle) (Fig. 5L). WM4257-vec tumors were also more sensitive to TN compared to WM4257-*MCL1* tumors, but with a lower magnitude of differences (Fig. S6E and S6F).

### Inhibition of MCL1 sensitizes DTN/DTV resistant PDXs

**Fig. 6:**
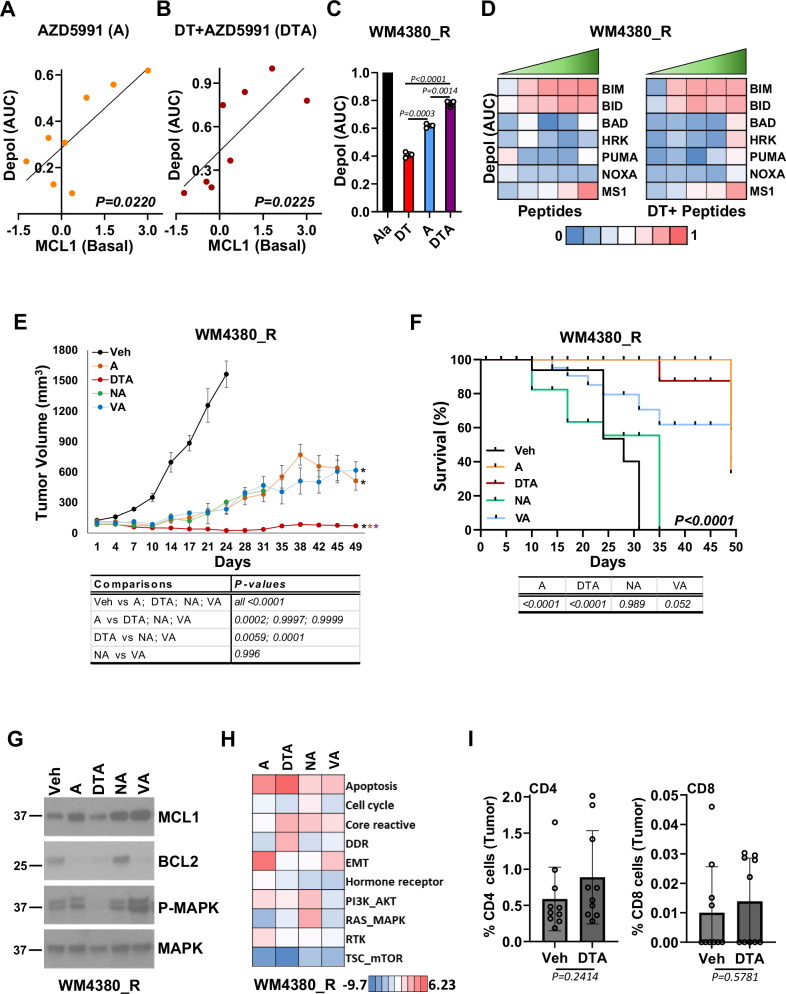
Inhibition of MCL1 counteracts resistance to BCL2 inhibition. Linear regression analysis of the association of MCL1 levels with mitochondrial membrane depolarizations induced by the MCL1i, AZD5991 (**A**), and its combination with DT, ‘DTA’ (**B**) in cells harvested from eight PDX models. Significance was determined using best-fit analysis and *P* values indicated on each graph. **C** Depolarizations induced by 25 µM Alamethicin ‘Ala’, 0.3 µM + 0.01 µM DT, 0.3 µM A and their combinations ‘DTA’ in WM4380_R cells. **D** Heatmaps showing depolarizations induced by BH3 peptides alone (1 µM, 10 µM, 50 µM, 100 µM, and 500 µM, left panel) and their combination with DT (0.3 µM + 0.01 µM, right panel) in WM4380_R. Heatmap scale at the bottom indicates highest and lowest range of depolarizations corresponding to positive control, Ala in red, and negative control, PUMA2A in blue. **E** Tumor growth of WM4380_R PDX implanted in mice and treated with vehicle (Veh, black), weekly once with 30 mg/kg single agent A (orange) or its combination with daily 30 mg/kg+1 mg/kg DT (DTA, red) or 75 mg/kg N (NA, green) or 75 mg/kg V (VA, blue). Data represents mean of ≥6 mice +/− SEM. Significance determined by two-way ANOVA and Tukey’s multiple comparisons tests; significant difference of any growth curve versus others (*P* < 0.05) is indicated by asterisks of the same color as the other growth curve and *P*-values shown below the graph. **F** Kaplan–Meier survival plot showing survival of mice (from *E*) treated with the indicated agents over a period of 49 days. Significance was determined by Log-rank test and *P*-values of treatments versus vehicle are shown below the graph. **G** Western blots of indicated proteins in WM4380_R tumor lysates from the tumor growth study in *E*. MAPK (ERK2) was used as a loading control. Molecular weight markers in kDa are indicated on the left of each blot. Results are from one experiment and two separate gels that were processed in parallel. **H** Differentially regulated protein signaling networks from RPPA analysis of WM4380_R tumor lysates. **I** IHC analysis of tumor**-**infiltrated CD4 and CD8 T cells shown as percentage of total positive staining cells in WM4380_R PDX tumors grown in CD34+ humanized mice treated with DTA. Data represents mean of ten random 1 mm^2^ measurements +/− SD in single mice, and significance determined by two-sided *t*-tests and *P*-values indicated below the graph.

Previous pre-clinical studies showed that combination treatment with MCL1 inhibitors (MCL1i) can improve the efficacy of BCL2i in hematological cancers^37,38^ and BRAFi/MEKi in solid cancers including melanomas^21,39,40^. However, it is not known whether MCL1i alone or in combination with BRAFi+MEKi could be effective against MAPKi treatment−progressed tumors from patients. BH3 profiling in eight BRAFi/MEKi-resistant PDXs treated with the MCL1i AZD5991 (abbreviated as A) or its combination with DT (DTA) showed that these treatments induced significantly higher mitochondrial depolarization in PDXs with high MCL1 expression (Fig. 6A, B). Additionally, BH3 profiling in WM4380_R, a high MCL1 expressing PDX (Fig. 2B) resistant to DTN/DTV (Fig S2E) showed significantly higher depolarization with A and DTA compared to DT (Fig. 6C). Among the BH3 peptides, the MCL1-specific MS1 peptide showed high depolarization along with BIM and BID (Fig. 6D, left panel), and 24 h DT treatment increased these effects (Fig. 6D, right panel), suggesting that MCL1i may sensitize high MCL1 expressing melanomas and may improve the efficacy of BRAFi+MEKi in melanomas with MCL1 mediated-resistance. Thus, we evaluated the anti-tumor activity of single agent A and its combination with N (NA), V (VA) or DT (DTA) in the WM4380_R PDX. Single agent A or its combinations with N (NA) or V (VA) significantly slowed tumor growth, but only combination treatment with DT (DTA) achieved tumor regression (Fig. 6E). Withdrawal of A, VA and DTA treatments from days 31−40 resulted in a small increase of tumor growth. Resumption of treatments (at day 41) decreased tumor growth in A and VA groups, but induced tumor regressions in the DTA group with no detectable tumors upon dissection at the end of the experiment (Fig. 6E). Combination of A with either N or V caused weight loss in mice, suggesting systemic toxicity, with NA causing the worst weight loss among all treatments. A Kaplan–Meier survival analysis with >15% weight loss or >1500 mm^3^ tumor size as survival endpoints showed that mice treated with A and DTA had significantly better survival compared to mice treated with Vehicle, NA or VA (Fig. 6F).

Western blotting analysis of the WM4380_R tumor lysates collected after seven days of treatment showed that DT, N, V or their combinations induced small increases of MCL1 expression; DT decreased BCL2, while N and V increased it (Fig S6G). Treatment with A and its combination with N (NA) or V (VA) increased MCL1, but its combination with DT (DTA) did not (Fig. 6G). Additionally, treatment with A alone and in combination with DT or V (but not N) decreased BCL2 (Fig. 6G). Finally, DT, DTN, DTV and DTA inhibited P-MAPK levels (Fig. 6G and S6G). Protein signaling analysis of RPPA proteomics data showed largest increase in apoptosis pathway and largest decrease in pro-survival TSC-mTOR pathway in DTA treated tumors compared to other treatments (Fig. 6H and Fig S6H). IHC analysis of tumors grown in NSG-SGM3 humanized mice showed that DTA treatment did not significantly alter CD4 and CD8 T cell infiltration into tumors compared to vehicle (Fig. 6I; spleen control in Fig S6I).

### MAPK pathway inhibition decreases cardiotoxicity of MCL1 inhibition

**Fig. 7:**
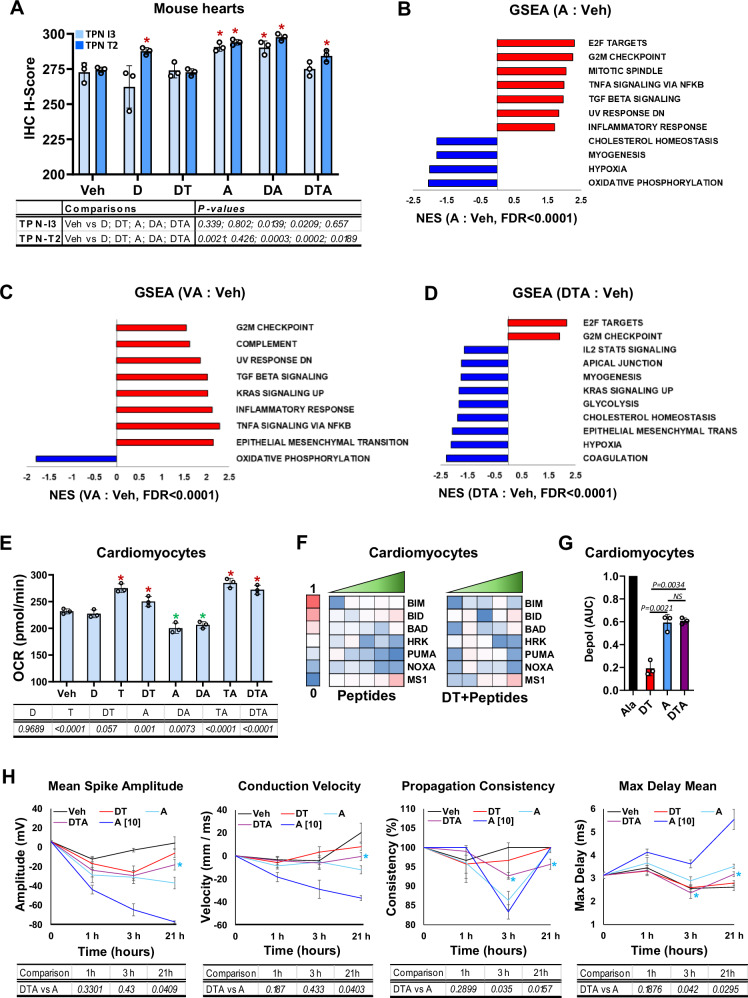
Effect of MCL1 and MAPK inhibition on cardiomyocytes and cardiac function. **A** IHC H-scores of Troponin I3, ‘TPN I3’ and Troponin T2, ‘TPN T2’ in mouse hearts harvested after treatment of mice with vehicle or the indicated treatments- daily once dosing of D and T (30 mg/kg D, 1 mg/kg T), and weekly once dosing of A (30 mg/kg). Data represents measurements from three mice per treatment +/− SD, and significance determined by two-sided t-tests and *P*-values indicated below the *x*-axis. GSEA analysis showing the most significantly altered (*FDR* = *0*) transcriptional networks in RNA of WM4380_R tumors after treatment with A (**B**), VA (**C**), or DTA (**D**). **E** Basal oxygen consumption rate (OCR) in human cardiomyocytes after 24 h treatment with 0.1 µM D, 0.03 µM T, 1 µM A or their combinations as shown. Data represents biological triplicates +/− SD; significance determined by two-sided *t*-tests and colored asterisks indicate significantly higher (red) or significantly lower (green) OCR versus vehicle, with *P*-values shown below graph. **F** Heatmaps showing depolarizations induced by increasing doses (1 µM, 10 µM, 50 µM, 100 µM, and 500 µM) of BH3 peptides alone (left panel) and their combination with DT (right panel) in human cardiomyocytes. Scale indicates highest and lowest range of depolarizations corresponding to positive control, Ala in red, and negative control, PUMA2A in blue, respectively. **G** Depolarizations induced by Ala, DT, A and DTA in human cardiomyocytes. Mimetic and peptide doses were as in Fig. 6C, D. Data is from biological triplicates +/- SD and significance determined by two-sided *t*-tests. **H** Cardiomyocytes in MEA plates were treated with DMSO vehicle, 0.1 µM + 0.03 µM DT, 1 µM A, their combination DTA, or with high dose 10 µM of A (dark blue line). MEA electrophysiological measurements were performed at 1 h, 3 h and 21 h, and results for the four indicated cardiac function tests were generated. Data represents biological triplicates +/− SEM, and significance determined by one-sided *t*-tests, with blue asterisks indicating significant differences between DTA and A treatments, and *P*-values are shown below respective graphs.

Recent clinical studies showed that treatment with MCL1i and its combination with BCL1i induced cardiotoxicity in patients (NCT03218683, NCT03465540, NCT02979366, NCT02992483). The weight-loss/survival data in Fig. 6F did not reveal overt toxicity in mice treated with A, possibly because it has ~25-fold lower binding affinity for mouse MCL1i than human (*Ki* for mouse MCL1: ~0.005 nM; human MCL1: ~0.13 nM). Nevertheless, IHC analysis of two established clinical biomarkers of cardiac toxicity, Troponin I3 and Troponin T2, showed a significant increase in both Troponins in the heart tissues of mice treated with A and DA for 15 days compared to vehicle controls (Fig. 7A). Interestingly, DT and DTA treatment did not increase either Troponin levels (Fig. 7A).

Inhibition or knockdown of MCL1 was previously shown to cause mitochondrial dysfunction in cardiomyocytes by altering mitochondrial membrane potential and inhibiting oxidative phosphorylation (OxPhos), resulting in cardiotoxicity^41,42^. GSEA of RNA-seq data from WM4380_R tumors revealed that long-term (30 days) treatments with A or VA inhibited OxPhos in addition to other transcriptional networks involved in cellular metabolism (Fig. 7B, C and Fig S7A, S7B). Interestingly, DTA treatment did not inhibit OxPhos, although it induced other similar transcriptional alterations as A and VA (Fig. 7D, Fig. S7C). As inhibition of MAPK pathway with BRAFi or MEKi activates OxPhos^43–45^, we tested whether DT opposes OxPhos inhibition by MCL1i and prevents mitochondrial dysfunction. OCR measurement as a surrogate for OxPhos showed that treatment with D, T or their combination for 24 h activated OCR in the *BRAF*^*V600*^-mutant WM4380_R cells (Fig. S7D). Treatment with single agent A inhibited OCR, but combining D and T with A (DA, TA and DTA) opposed OCR inhibition by A (Fig. S7D), suggesting that MAPK inhibition indeed opposes OxPhos downregulation by MCL1 inhibition, and may counteract MCL1i-induced increase of cardiac troponins seen in Fig. 7A.

Based on the above findings in the BRAF-mutant melanoma PDX and cells, we evaluated whether OxPhos upregulation by MAPKi opposes OxPhos downregulation by MCL1i in cardiomyocytes and prevent cardiac toxicity. Treatment of human cardiomyocytes with D did not alter OCR in these BRAF-wild type cells, indicative of a lack of MAPK inhibition by this *BRAF*^*V600*^ mutant-specific inhibitor (Fig. 7E). However, single agent T activated OCR, indicative of OxPhos activation via MAPK inhibition by T. Treatment with A inhibited OCR, indicative of OxPhos inhibition by A. Combination of D with A (DA) did not prevent OCR inhibition, but the combination of either T or DT with A (TA and DTA) prevented OCR inhibition (Fig. 7E).

Cell viability assessments using trypan blue staining after treatment with A revealed a small decrease in cardiomyocyte viability over a period of 72 h, but its combination with DT did not significantly alter viability (Fig S7E). BH3 profiling showed that high doses of BID and the MCL1-specific MS1 peptide induced some depolarization in these cardiomyocytes, but combination with DT led to a decrease in depolarization at lower doses of MS1 (Fig. 7F). Treatment with A increased depolarization significantly compared to DT, but DTA did not further increase depolarization in the cardiomyocytes (Fig. 7G).

Finally, we evaluated cardiomyocyte function in monolayers of electrically active human cardiomyocytes that generate uniform rhythmic pulses (heartbeats). We performed in vitro electrophysiological analysis of ‘mean spike amplitude’ and ‘conduction velocity’ which measure average change in membrane potential leading to rhythmic electrical conduction. We additionally measured ‘propagation consistency’ of electrical impulses and ‘max delay mean’ time from impulse to cell’s mechanical response. Treatment-induced alterations in these parameters over time would identify impaired contractility, arrhythmia and cardiac dysfunction. We performed treatments with the indicated concentrations of A, DT, DTA and included as a positive control, a lethal high dose of A (10 µM) that potently inhibits cardiomyocyte function (Fig. 7H). Electrophysiological measurements following treatments showed that 21 h treatment with single agent A significantly inhibited ‘mean spike amplitude’ and ‘conduction velocity’, which are critical to cardiomyocyte function (Fig. 7H). Combination with DT (DTA) decreased their inhibition by A. Treatment with A also significantly inhibited ‘propagation consistency’ signal within 3 h, while combination with DT (DTA) decreased this inhibition. Interestingly, while inhibition of ‘propagation consistency’ by A was completely reverted after overnight incubation, it was prolonged with DTA treatment. Finally, ‘max delay mean’ was significantly increased by treatment with A, while DTA counteracted it. Altogether, these results show that MCL1 inhibition increased cardiac toxicity markers in mouse hearts, decreased OxPhos and inhibited cardiomyocyte function, all of which were counteracted significantly by MAPK inhibition.

## Discussion

Therapeutic options are limited for metastatic melanoma patients with progressive disease following treatment with standard-of-care immune and targeted therapies, creating a need for personalized approaches to understand and counteract resistance. Our analysis of melanoma patient responses to BRAFi+MEKi treatments implicated elevated tumor BCL2 family proteins with resistance, similar to previous analysis of single-agent BRAFi^13^. A unique collection of melanoma PDX models, mostly established from tumors exhibiting acquired resistance to BRAFi or BRAFi+MEKi, facilitated our extensive investigation of BCL2 and BCL-xL inhibition in this setting, with a goal to identify a biomarker-guided subset of patients who could benefit from these treatments. Our results showed that single agent V (BCL2i) or N (BCL2i and BCL-xLi) do not induce tumor stasis or regression in BRAFi+MEKi-resistant melanoma PDXs. However, combining BRAFi+MEKi with either BH3 mimetic induced potent tumor growth inhibition and even regressions in a subset of PDXs with high basal BCL2 and low basal MCL1. Although our results suggest that DTV was slightly more effective against melanomas with high basal BCL2 levels compared to those with high BCL-xL, DTN was generally effective against both types and potentially superior. This supports accumulating clinical evidence of resistance to BCL2-specific inhibitors due to treatment-induced upregulation of BCL-xL, renewing the need for BCL-xL targeting^17^. Notably, our analysis of TCGA data also show that both BCL2 and BCL-xL are expressed at high levels in melanoma compared to other tumor types. There is therefore a strong rationale to inhibit both BCL2 and BCL-xL, while acknowledging the risk that BCL-xL inhibition may result in thrombocytopenia, as has been observed in high-dose single agent and combination regimens with N^46,47^. However, dose modification of N in some combination studies, including a phase II study with a JAK1/2 inhibitor in myelodysplasia patients, showed manageable toxicity^47,48^, resulting in advancement of the treatment to phase III (NCT04472598, NCT04468984). In melanoma, initial results from a randomized phase II clinical trial in treatment-naïve BRAF-mutant stage IV patients revealed that the triple combination treatment with DTN was well tolerated and caused no increase in the rate of ≥ Grade 3 thrombocytopenia compared to treatment with DT alone. Treatment with DTN also improved survival compared to DT only, although it did not significantly improve response rates or progression-free survival^36^. However, this trial did not use a biomarker-guided patient-selection strategy for DTN treatment, and our unpublished analysis of several DTN treatment-progressed patient samples from the trial identified an increase of MCL1 protein in the patient tumors (not shown, manuscript in preparation). Thus, our results suggest that a personalized biomarker-guided strategy targeting BCL-2 and BCL-xL could be clinically beneficial in BRAFi/MEKi-resistant melanoma patients with high tumor BCL2 and low MCL1.

Unlike BCL2, MCL1 levels were not uniformly upregulated by BRAFi/MEKi treatment in our melanoma patient tumor cohort, and this heterogeneity was also observed in the PDXs treated with DT or DTN/V. However, high basal MCL1 and treatment induced MCL1 in the PDXs were significantly associated with resistance to DTN/V. Overexpression studies functionally confirmed the role of MCL1 in resistance to DTN/V. Additionally, treatment with single agent MCL1i induced potent growth inhibition of a MCL1 over-expressing PDX, and its combination with BRAFi+MEKi induced its complete regression. Together, these results suggest that BRAFi+MEKi+MCL1i is a promising therapeutic option for MCL1 overexpressing melanomas that are resistant to BRAFi+MEKi -/+ BCL2i.

Recent phase I clinical trials in hematological cancer patients with some MCL1i were prematurely stopped or are on-hold due to cardiotoxicity with MCL1i and its combinations with BCL2i. Cardiotoxicity could potentially be mitigated using rational strategies like limited or intermittent dosing, although such strategies could reduce in vivo PD/PK, resulting in sub-optimal anticancer effect. In this study, we found that BRAFi+MEKi treatment counteracted mitochondrial OxPhos inhibition and prevented cardiac troponin activation by MCL1i, and restored cardiomyocyte functionality. This suggests an unexpected strategy to decrease cardiotoxicity of MCL1i, and is reminiscent of the distinct clinical benefit of combining MEKi with BRAFi to decrease cutaneous toxicity induced by single agent BRAFi^3,49^. In a broader context, it is evocative of titrating doses of poisons with antidotes to counteract toxicity of either reagent. However, our results are from studies using mice and in vitro human cardiomyocytes, and while toxicity from BRAFi+MEKi treatments in patients is well-understood and well-managed^50^, the safety of MCL1i combination with BRAFi+MEKi will still need to be evaluated in a clinical study.

A limitation with iPSC-derived cardiomyocytes used in our study is that they may not faithfully represent adult human cardiomyocyte metabolism with respect to substrate preference for oxidative phosphorylation^51^. While our studies are not necessarily impacted by substrate preference, adult cardiomyocytes could be tested as an alternative, although those may have other limitations like lack of excitability in 2D cultures. Nevertheless, with cardiac troponins serving as highly sensitive early markers of MCL1i cardiotoxicity, evaluating this toxicity in a clinical trial of BRAFi+MEKi+MCL1i treatments is feasible. Alternatively, recent studies with CDK7/9 inhibitors suggest that these compounds indirectly inhibit MCL1 expression and may be safer than MCL1i^52,53^. Early clinical trial results suggested manageable toxicity^54,55^, and we are investigating melanoma tumor growth inhibition and potential cardiotoxicity of these inhibitors in a separate study. In addition to cardiotoxicity with MCL1i, there are also concerns that long-term BCL2 inhibitor treatment may potentially disrupt immune cell function^32^. While the PDX models used in our study did not permit evaluation of the treatments on host immune cells, particularly TILs, our results in humanized mice showed that BCLi2/BCL-xLi and their combination with MAPKi did not adversely alter CD4+ and CD8 + T cell infiltration into tumors. Consistent with this, recent studies also suggested that BCL2i can in fact stimulate anticancer immune cells and improve patient outcomes^33–35,56^.

In summary, using a large collection of molecularly characterized melanoma PDXs and patient samples, we found that targeting BCL2 family anti-apoptotic proteins may help to overcome acquired resistance to standard-of-care BRAFi+MEKi treatment. Molecular and functional assessments of the PDXs identified baseline high BCL2 and low MCL1 as potential predictive biomarkers of benefit from this strategy. These results can be potentially translated into personalized strategies for BRAF-mutant melanoma patients with BRAFi/MEKi−refractory disease, for whom new therapeutic options are critically needed.

## Methods

The research in this study complies with all relevant ethical regulations of M.D. Anderson Cancer Center.

### Melanoma patient samples

Melanoma tumor samples from patients prior to or post-treatment with Vemurafenib (Vem) or Dabrafenib+Trametinib (DT) and a tissue microarray of stage IV melanoma patient tumors were retrospectively requested from the M.D. Anderson Melanoma Informatics, Tissue Resource, and Procurement Core (MelCore). All samples and associated data were collected under Institutional Review Board (IRB) approved protocol and in accordance with the 1964 Declaration of Helsinki and its later amendments. Assigned sex of the patients was collected under informed consent and de-identified sex disaggregated patient data are shown in Fig. S1I and S5B.

### Melanoma Patient-derived xenografts (PDXs)

The melanoma PDXs used in this study and listed in Fig. 2B meet or exceed the minimal information standard for PDXs (PDX-MI) (PMID: 29092942). Patient data including clinical responses to treatments, driver mutations, assigned sex and tumor samples for PDX generation were collected under informed consent at the original source. Patient data was de-identified for Protected Health Information (PHI) under the Health Insurance Portability and Accountability Act of 1996 (HIPAA). The ages of patients within this cohort ranged between 37 and 83 years at biopsy. Basal gene expression and basal protein expression profiles, whole exome sequencing as well as the clinical information for these PDXs have been reported earlier^23,57^. PDXs were maintained as frozen stocks in 10% DMSO and 50% fetal bovine serum (FBS) in RPMI-1640 media.

### Inhibitors and treatments

Trametinib, Dabrafenib, Navitoclax, Venetoclax, and AZD5991 were obtained from the National Cancer Institute (NCI) through the CTEP and DCTD programs (https://ctep.cancer.gov/). For in vitro treatments, all compounds were dissolved in DMSO and used at the indicated concentrations in the experiments. For in vivo treatments, the following amounts of the compounds were infused into 1 kg of Teklad Global standard natural ingredient 18% Protein Rodent Diet with 4,100 ppm Uniprim: 150 mg dabrafenib (D) and 5 mg trametinib (T); 375 mg Navitoclax (N) or Venetoclax (V); or their mixtures to make the respective combinations of DTN or DTV. Daily drug dosing from chow infused diets results in average ingested doses of 30 mg/kg of D, 1 mg/kg of T, 75 mg/kg of N or V. AZD5991 (A) was dissolved in 30% HPBCD (hydroxy-propyl-beta-cyclodextrin) in water adjusted to pH 9.0-9.5 with 1 M Meglumine, and freshly prepared one day prior to dosing. Treatments with AZD5991 were performed once a week using tail vein intravenous injections to achieve a dose of 30 mg/kg per animal. All drug dosing for tumor growth inhibition studies were determined after initial testing of targeted and toxicological effects of each compound and their combinations.

### Generation of PDX tumors in mice, therapeutic treatments and sample collections

Mice experiments were approved by the M.D. Anderson Institutional Animal Care and Use Committee (IACUC). Mice were housed in vivarium set to 22.2 °C temperature, 45% relative humidity, and 12 h light/dark cycles. PDX propagation and sub-cutaneous (s.c.) tumor growth inhibition studies in gender-matched NOD.Cg-Prkdc Il2rg/SzJ (NSG) mice have been described previously^23^. When tumor volumes reached approximately 100 mm³, mice were randomized into specific drug treatment groups (up to 10 mice per group) prior to treatments. Tumor volumes and animal weights were simultaneously recorded every three days. For molecular studies, three mice per group were euthanized for tumor collection at either an early time point (3-7 days after start of treatment) or at the end of the tumor growth study. Mice were euthanized if they exhibited physical distress or weight loss ≥20% body weight or if tumor sizes exceeded 2000 mm^3^, as permitted by the M.D. Anderson IACUC. In instances where the tumor sizes unexpectedly exceeded the measurement limit in the next measurement compared to the previous measurement, those next measurements were removed from analysis and mice euthanized. In some long-term treatment studies, mice with complete tumor regressions were put on short periods of drug holidays as indicated, and/or followed by prolonged (80–100 days) treatments to evaluate long-term effects. For cardiac toxicity studies, mice hearts were collected following treatments for the indicated times. For assessing treatment effects on tumor immune cell infiltration, PDX tumors were grown s.c. in NSG-SGM3 mice that were humanized with human CD34+ hematopoietic stem cell engraftment (Jackson Laboratory). Tumors and spleens were collected at the indicated times following treatments.

### Lentiviral transduction of PDX-derived cells, generation of cell clones and in vivo tumor growth studies

Melanoma PDX tumors grown in NSG mice were harvested to isolate single cell populations as described before^23^. Cells were seeded in 6 well plates and infected with 10^7^ CFU lentiviral particles expressing human *MCL1* (NM_021960) in a pLenti-C-Myc vector (OriGene cat # RC200521L1V) or empty control in the same vector using standard lentiviral infection with 10 µg/ml polybrene. After 6 h incubation, the cell infection media was replaced with cell growth media containing 2 µg/ml puromycin antibiotic to select infected cells over a period of four weeks. Cell clones were evaluated for expression of exogenous MCL1 protein using western blotting, and clones expressing high exogenous MCL1 were separated and maintained in 0.2 µg/ml puromycin containing media. Clones showing consistent overexpression of MCL1 were used in cell/tumor growth studies. Sub-cutaneous xenograft tumors from cell clones were generated by injecting the MCL1 or control vector expressing cells (3 × 10^6^ cells/animal) in the right flank of NSG mice. Mice randomization, drug treatments, tumor measurements and harvests were performed as described above for the PDX tumors.

### Nucleic acid purification and sequencing

DNA and RNA were purified from snap-frozen PDX tumors using QIAamp DNA mini kit (Qiagen) and mirVana mRNA isolation kit (Invitrogen) respectively and sequenced at the Broad Institute Genomics core facility (Boston, MA). PCR-Free Human WGS − 60x v2 was used for whole genome DNA sequencing, with sample preparation utilizing custom Broad indices (IDT) and Kapa Biosciences HyperPrep library construction kit, sequencing (Illumina 2x150bp reads), read de-multiplexing, aggregation and alignment (Bwa-Mem). RNA-seq was performed using Illumina TruSeq strand-specific protocol with 151 bp paired end reads for sequencing, with a sequence coverage to 50 M paired end reads.

### Protein analysis

Whole cell lysate preparation from cell lines, tumors and other tissues, and quantification of proteins using BCA protein assay were previously described^44^. Protein lysates were denatured for western blotting and Reverse Phase Protein Array (RPPA). Western blotting was performed using standard methods and RPPA analysis was performed at the MDACC Functional Proteomics Core Facility. RPPA data was analyzed as described previously^43^. Antibodies, their vendors and catalog numbers used for western blotting and RPPA are listed in the RPPA core website (www.mdanderson.org/research/research-resources/core-facilities/functional-proteomics-rppa-core.html). Primary antibodies for western blotting are MCL1 (CST 39224), BCL2 (CST 15071), Phospho-MAPK (ERK1/2) (CST 4370), MAPK (ERK2 (SC-1647) and GAPDH (CST 97166) used at a dilution of 1:1000 in 5% solution of BSA in TBS-T buffer.

### Immunohistochemistry staining

Xenograft tumor tissues and mouse hearts harvested from mice were formalin-fixed, paraffin-embedded, serially sectioned into 5 µm slices and transferred to glass slides for staining. Human melanoma tumor samples from patients prior to and post-treatments were also similarly sectioned for staining. Immunohistochemical staining of tissue sections was performed on a Leica Bond RXm autostainer using the following antibodies: MCL1 (CST 39224), BCL2 (CST 15071), BCL-xL (CST 2764), Troponin I3 (21652-1-AP), Troponin T2 (15513-1-AP), CD4 (CST 25229) and CD8 (CST 98941). Slides were scanned at 20X magnification using Aperio Scanscope (Aperio/Leica Microsystems). Immunohistochemistry staining of extra- and intra-cellular features was interpreted concurrently with H&E staining and positive cells for each marker were quantified using Aperio ImageScope software in five random areas (1 mm^2^ each). Stained slides were scored by a board-certified pathologist and given an H-score based on percentage and intensity of positivity ranging from 0 to 3^58^. H-scores were calculated using the formula [(%tumor cells 1 + ) x 1] + [(%tumor cells 2 + ) x 2] + [(%tumor cells 3 + ) x 3) as described before^59^. For immune cells, the Nuclear-v9 algorithm in ImageScope was customized by altering nuclear size detection to include smaller nuclei (10 µm^2^ instead of the standard 20 µm^2^) and removing background artifacts by adjusting “remove light objects” to 0.01 from 0, and quantification was performed in up to ten random areas.

### Analysis of mitochondrial oxidative phosphorylation

For PDXs, tumors from treatment mice were harvested to isolate tumor cells, and seeded in 96 well Seahorse XF plates (1.5 × 10^4^ cells per well) overnight in presence of their respective treatments, followed by performing bioenergetics stress tests in a Seahorse XF analyzer as previously described^43^. In addition to measuring basal oxygen consumption rate (OCR), Oligomycin (1.5 µM), FCCP (0.5 µM), Rotenone (0.5 µM) + Antimycin (0.5 µM) treatments were administered to assess oxygen used for mitochondrial ATP synthesis, maximal respiratory capacity and mitochondrial oxygen consumption by the cells, respectively. For assessing treatment-induced OCR alterations in human cardiomyocytes, immortalized human cardiomyocytes were cultured in PriGrow I media with 5% FBS in PriCoat flasks (Applied Biological Materials) for one week prior to transferring the cells to 96 well Seahorse XF plates. The cells were then incubated with the indicated treatments for 24 h, followed by measurement of basal OCR at 37 °C in serum-free media. Seahorse data was normalized against cell numbers.

### BH3 profiling

Single cell populations of melanoma cells were isolated from s.c. PDXs harvested from NSG mice as described before^23^. A JC1 plate-based BH3 profiling assay was performed as described^60^. Cells were strained using a 40-micron strainer and permeabilized for five minutes with JC-1 cell staining solution consisting of digitonin and mitochondrial experimental buffer^61^ prior to seeding them in black 384-well plates at ~5 × 10^3^ cells per well in triplicates. Prior to cell addition, BH3 peptides and mimetics treatments were added to the plates at indicated doses. The cell-loaded plates were incubated at 32 °C for an additional five minutes and placed in an Infinite M1000 Pro plate reader set to record fluorescence (545 nm excitation, 590 nm emission) every 10 min for a total time of 3 h. For determining BRAFi+MEKi effects, cells were treated with 0.3 *µ*M Dabrafenib + 0.01 *µM* Trametinib for 24 h prior to permeabilization and addition to the plates. Area under the curve (AUC) for every well was determined from the fluorescence reads and depolarization was calculated using the formula: *Depolarization* = *(AUC*_*Sample*_*–AUC*_*Alamethicin*_*)/(AUC*_*Control*_*–AUC*_*Alamethicin*_*)*, where *Sample* is cells treated with either BH3 peptide or mimetic, *Control* is vehicle control PUMA2A or DMSO. Alamethicin (25 µM) served as the depolarized positive control, and depolarization of samples normalized against depolarization of alamethicin were represented as heatmaps or column graphs. For BH3 profiling of human cardiomyocytes, the cells were cultured as described for the bioenergetics stress test, followed by JC-1 staining, seeding (3 × 10^3^ cells/well), and fluorescence measurements.

### Cardiomyocyte electrophysiology analysis

This study was performed at the Microphysiological Leads Optimization and Toxicity Screening (MLOTS) core division of the Institute of Biosciences and Technology, Texas A&M University College of Medicine. Human induced pluripotent stem (iPS) cardiomyocytes (FujiFilm Cellular Dynamics, Inc) were cultured in iCell cardiomyocyte medium (M1003, FujiFilm) for 8 days in a 96-well Multi-Electrode Array (MEA) plate containing 8 electrodes per well, followed by treatment with test and reference compounds. Cardiomyocyte activity was recorded using the Maestro-361 multi-well system with AxIS software (AxIS Suite 3.12) from Axion Biosystems. All MEA recordings were conducted at 37 °C in culture medium without perfusion. Baseline recordings, as well as 1 h, 3 h and 21 h post dosing data was recorded, and baseline-normalized data was plotted in line graphs.

### Statistics and reproducibility

GraphPad Prism and Microsoft Excel were used for statistical analysis and for generating graphical presentations. A minimum of three biological replicates were used in all experiments with sample sizes indicated in figure legends and repeated as required for reproducibility. For tumor growth inhibition studies, mice were randomized into treatment groups and investigators were blinded to allocation and outcome assessments. Sample sizes chosen to provide 80% power at α = 0.05 to detect at least a 30% difference between treatment groups. No data was excluded from analysis. Protein signaling pathways from RPPA data were generated as described earlier^28^ after batch corrections using MBatch (https://bioinformatics.mdanderson.org/public-software/mbatch/). Supervised hierarchical clustering of protein pathways in treatments versus vehicle samples was performed using Pearson’s correlation coefficient in Gene Cluster 3.0, and heatmaps were generated using Gene Treeview software^44^. RNA-seq results were analyzed by Gene Set Enrichment Analysis (GSEA) identifying normalized enrichment scores (NES) in treatments versus vehicle or between treatments^62^.

### Reporting summary

Further information on research design is available in the Nature Portfolio Reporting Summary linked to this article.

## Supplementary information

Supplementary Information

Peer Review file

Reporting Summary

## Source data

Source Data

## Data Availability

Deidentified RNAseq and DNA mutation data of melanoma PDXs in the study have been deposited in NCBI GEO (https://www.ncbi.nlm.nih.gov/geo/query/acc.cgi, GEO accession GSE298507). TCGA RPPA data is available on the TCGA website (https://gdc.cancer.gov/subject-tag/tcga). Antibodies used for RPPA are listed on the MDACC Functional Proteomics RPPA core website (https://www.mdanderson.org/research/research-resources/core-facilities/functional-proteomics-rppa-core/education-and-references.html). All other data is included in the source data file provided with this paper. Any additional information can be requested from the authors. Source data are provided with this paper.
