# Supplementary material for: Personalized targeting of BCL2 family proteins overcomes acquired resistance to BRAF-MEK inhibitors in preclinical melanoma
Source: Nat Commun. 2026 Jun 26;17:7582. doi: 10.1038/s41467-026-74691-9 (PMC13421458; doi:10.1038/s41467-026-74691-9)
Supplement: Supplementary file 1 — Supplementary Information [file 41467_2026_74691_MOESM1_ESM.pdf]

Figure S1

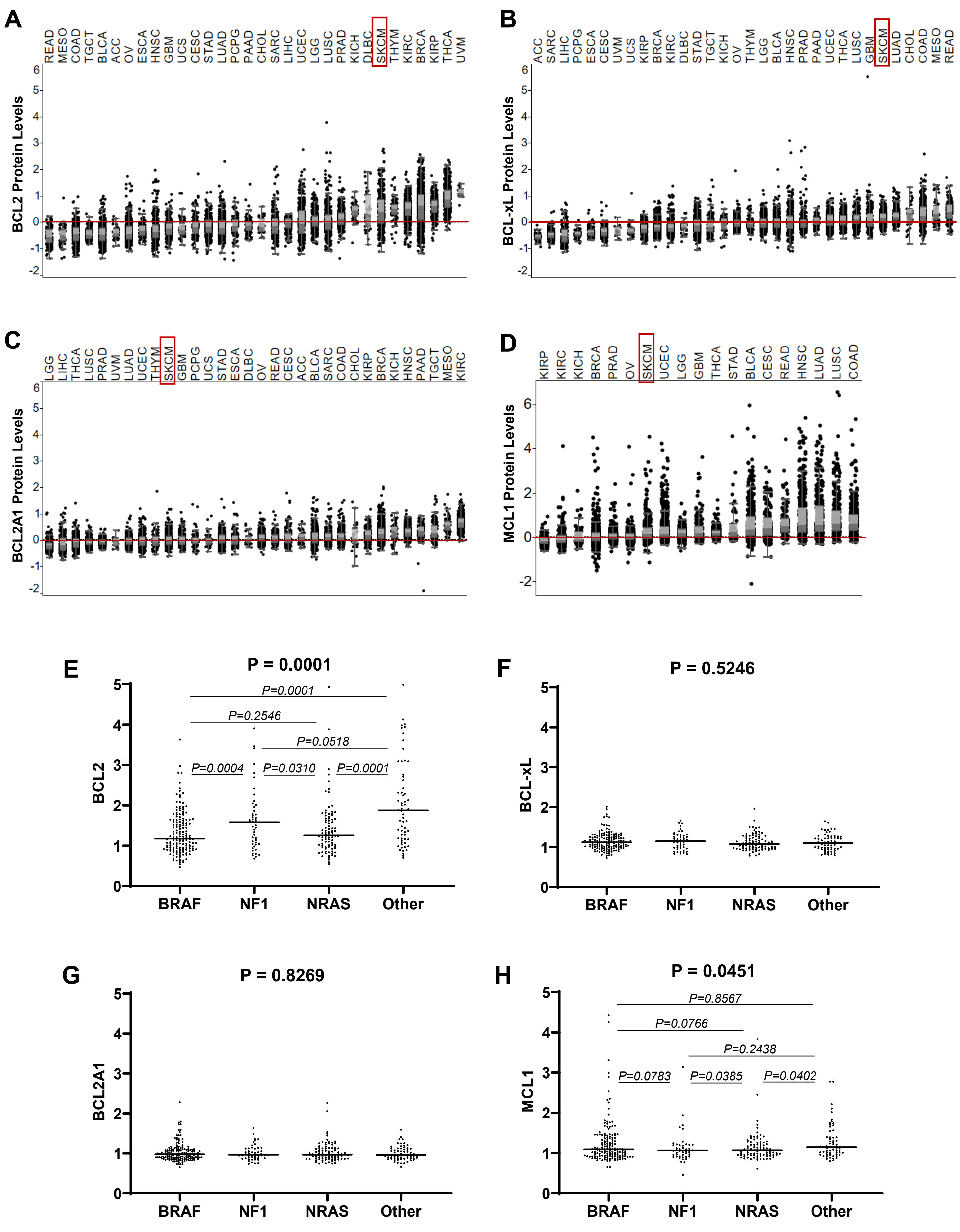

A
READ MESO COAD TGCT BLCA ACC OV ESCA HNSC GBM UCS CESC STAD LUAD PCPG PAAD CHOL SARC LIHC UCEC LGG LUSC PRAD KICH DLBC SKCM THYM KIRC BRCA KIRP THCA UVM
BCL2 Protein Levels
B
ACC SARC LIHC PCPG ESCA CESC UVM UCS KIRP BRCA KIRC DLBC STAD TGCT KICH OV THYM LGG BLCA HNSC PRAD PAAD UCEC THCA LUSC GBM SKCM LUAD CHOL COAD MESO READ
BCL-xL Protein Levels
C
LGG LIHC THCA LUSC PRAD UVM LUAD UCEC THYM SKCM GBM PCPG UCS STAD ESCA DLBC OV READ CESC ACC BLCA SARC COAD CHOL KIRP BRCA KICH HNSC PAAD TGCT MESO KIRC
BCL2A1 Protein Levels
D
KIRP KIRC KICH BRCA PRAD OV SKCM UCEC LGG GBM THCA STAD BLCA CESC READ HNSC LUAD LUSC COAD
MCL1 Protein Levels
E
P = 0.0001
P=0.0001
P=0.2546
P=0.0518
P=0.0004
P=0.0310
P=0.0001
BCL2
BRAF NF1 NRAS Other
F
P = 0.5246
BCL-xL
BRAF NF1 NRAS Other
G
P = 0.8269
BCL2A1
BRAF NF1 NRAS Other
H
P = 0.0451
P=0.8567
P=0.0766
P=0.2438
P=0.0783
P=0.0385
P=0.0402
MCL1
BRAF NF1 NRAS Other

I

Patient #	Sex	Treatment	Pre (BCL2)	Post (BCL2)	Pre (MCL1)	Post (MCL1)
1	F	DT	115	250	60	95
2	M	DT	180	300	0	215
3	F	Vem	0	225	40	180
4	M	Vem	170	285	60	10
5	M	DT	100	–	85	–
6	M	DT	250	–	190	–
7	M	DT	–	270	–	205
8	M	Vem	–	200	–	280
9	M	Vem	–	140	–	180

J

Fig 2C (WM3903_R)	
Tukey's multiple comparisons test	P Value
Veh vs. DT	<0.0001
Veh vs. DTN	<0.0001
Veh vs. N	0.0005
Veh vs. V	0.0957
Veh vs. DTV	<0.0001
DT vs. DTN	<0.0001
DT vs. N	0.1331
DT vs. V	0.0004
DT vs. DTV	0.2026
DTN vs. N	<0.0001
DTN vs. V	<0.0001
DTN vs. DTV	0.0026
N vs. V	0.53
N vs. DTV	0.0002
V vs. DTV	<0.0001

Fig 2D (WM4262_R)	
Tukey's multiple comparisons test	P Value
Veh vs. DT	<0.0001
Veh vs. DTN	<0.0001
Veh vs. N	<0.0001
Veh vs. V	<0.0001
Veh vs. DTV	<0.0001
DT vs. DTN	0.8521
DT vs. N	0.9354
DT vs. V	0.9681
DT vs. DTV	0.0795
DTN vs. N	0.2917
DTN vs. V	0.3716
DTN vs. DTV	0.6326
N vs. V	>0.9999
N vs. DTV	0.005
V vs. DTV	0.0081

Fig 2E (WM3960_R)	
Tukey's multiple comparisons test	P Value
Veh vs. DT	<0.0001
Veh vs. N	<0.0001
Veh vs. V	0.0217
Veh vs. DTN	<0.0001
Veh vs. DTV	<0.0001
DT vs. N	0.0921
DT vs. V	<0.0001
DT vs. DTN	0.6795
DT vs. DTV	0.3839
N vs. V	0.1922
N vs. DTN	0.001
N vs. DTV	0.0002
V vs. DTN	<0.0001
V vs. DTV	<0.0001
DTN vs. DTV	0.9972

Fig 2F (WM4070_R)	
Tukey's multiple comparisons test	P Value
Veh vs. DT	0.1565
Veh vs. DTN	<0.0001
Veh vs. N	0.1987
Veh vs. V	0.2949
Veh vs. DTV	<0.0001
DT vs. DTN	<0.0001
DT vs. N	0.0001
DT vs. V	0.0004
DT vs. DTV	<0.0001
DTN vs. N	<0.0001
DTN vs. V	<0.0001
DTN vs. DTV	0.9908
N vs. V	>0.9999
N vs. DTV	<0.0001
V vs. DTV	<0.0001

Figure S1: Anti-apoptosis BCL2 protein family expression in melanoma tumors. (A-D) Box scatter plots showing expression of BCL2 *(A)*, BCL-xL *(B)*, BCL2A1 *(C)* and MCL1 *(D)* proteins determined by RPPA in 32 different tumor types represented in the TCGA. RPPA data for MCL1 was available for 19 tumor types. Melanomas are notated as SKCM in red boxes. **(E-H)** Median scatter plots showing the expression of BCL2 (*E*), BCL-xL *(F)*, BCL2A1 *(G)* and MCL1 *(H)* in melanoma TCGA tumors with the three main driver mutations BRAF, NF1, NRAS or Other driver mutations. Significance of differences (*P<0.05*) assessed using one-way ANOVA between all groups, and post-hoc t-tests between individual groups. **(I)** Sex-disaggregated results of pre- and post-treatment BCL2 and MCL1 protein levels shown as H-scores in melanoma patient tumors. **(J)** P-values of treatment induced alterations between tumor growth curves from *Fig 2C-F.*

Figure S2

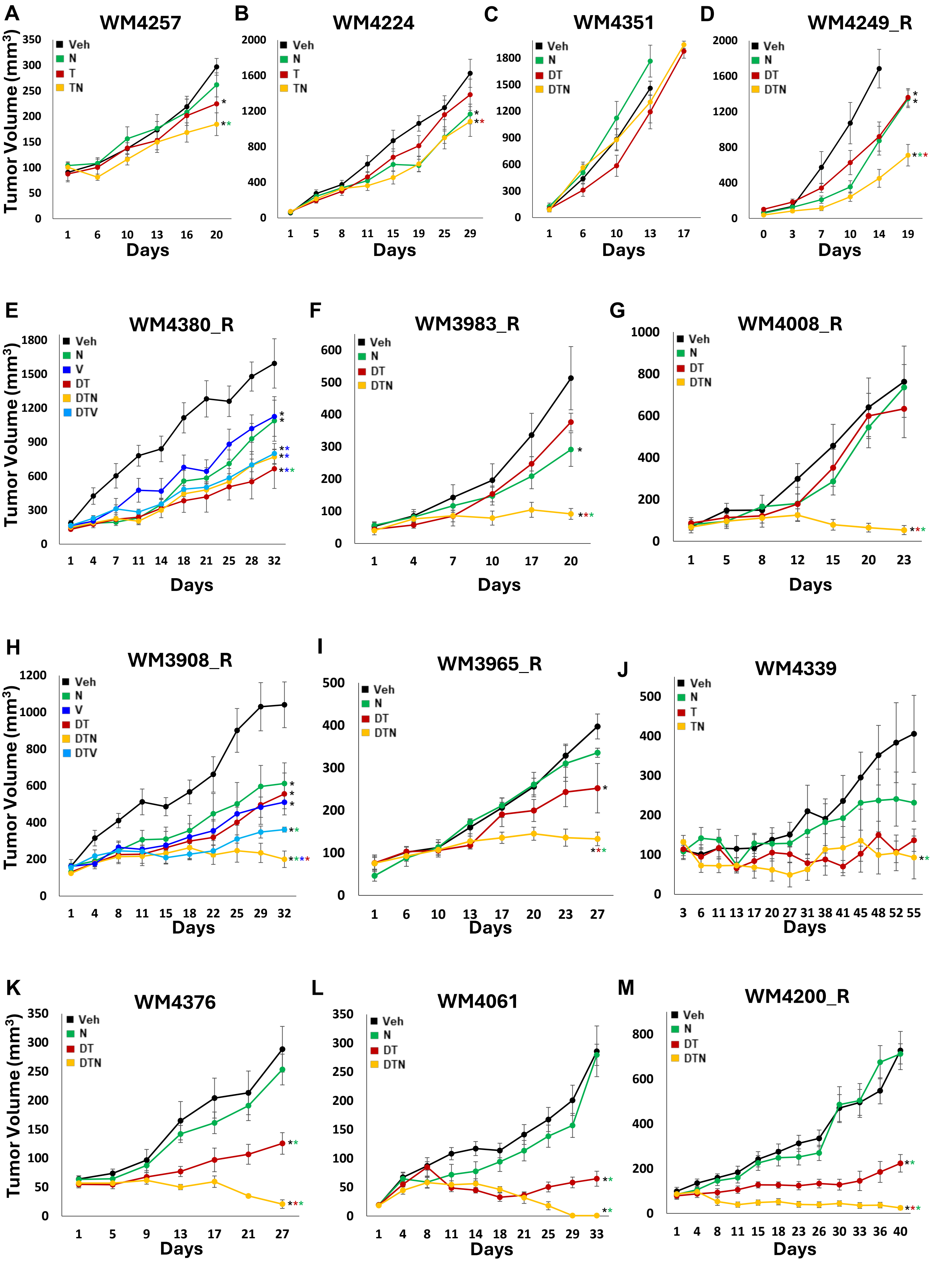

A
WM4257
Tumor Volume (mm³)
350 300 250 200 150 100 50 0
Veh
N
T
TN
1 6 10 13 16 20
Days
B
WM4224
2000 1600 1200 800 400 0
Veh
N
T
TN
1 5 8 11 15 19 25 29
Days
C
WM4351
1800 1500 1200 900 600 300 0
Veh
N
DT
DTN
1 6 10 13 17
Days
D
WM4249_R
2000 1600 1200 800 400 0
Veh
N
DT
DTN
0 3 7 10 14 19
Days
E
WM4380_R
Tumor Volume (mm³)
1800 1500 1200 900 600 300 0
Veh
N
V
DT
DTN
DTV
1 4 7 11 14 18 21 25 28 32
Days
F
WM3983_R
600 500 400 300 200 100 0
Veh
N
DT
DTN
1 4 7 10 17 20
Days
G
WM4008_R
1000 800 600 400 200 0
Veh
N
DT
DTN
1 5 8 12 15 20 23
Days
H
WM3908_R
Tumor Volume (mm³)
1200 1000 800 600 400 200 0
Veh
N
V
DT
DTN
DTV
1 4 8 11 15 18 22 25 29 32
Days
I
WM3965_R
500 400 300 200 100 0
Veh
N
DT
DTN
1 6 10 13 17 20 23 27
Days
J
WM4339
500 400 300 200 100 0
Veh
N
T
TN
3 6 11 13 17 20 27 31 38 41 45 48 52 55
Days
K
WM4376
Tumor Volume (mm³)
350 300 250 200 150 100 50 0
Veh
N
DT
DTN
1 5 9 13 17 21 27
Days
L
WM4061
350 300 250 200 150 100 50 0
Veh
N
DT
DTN
1 4 8 11 14 18 21 25 29 33
Days
M
WM4200_R
800 600 400 200 0
Veh
N
DT
DTN
1 4 8 11 15 18 23 26 30 33 36 40
Days

Figure S2: Effect of BCL2 inhibitor combinations with BRAF and MEK inhibitors on PDX tumor growth. PDXs were grown as sub-cutaneous tumors in the left flank of NSG mice and treated as follows- **(A)** WM4257 treated with vehicle (Veh), 75 mg/kg Navitoclax (N), 1 mg/kg Trametinib (T) or the combination of T with N (TN), **(B)** WM4224 treated with Veh, N, T or TN, **(C)** WM4351 treated with Veh, N, 30 mg/kg Dabrafenib + 1 mg/kg Trametinib (DT) or the combination of DT with N (DTN), **(D)** WM4249_R treated with Veh, N, DT or DTN, **(E)** WM4380_R treated with Veh, N, 75mg/kg Venetoclax (V), DT, DTN, or the combination of DT with V (DTV), **(F)** WM3983_R treated with Veh, N, DT or DTN, **(G)** WM4008_R treated with Veh, N, DT or DTN, **(H)** WM3908_R treated with Veh, N, V, DT, DTN or DTV, **(I)** WM3965_R treated with Veh, N, DT or DTN, **(J)** WM4339 treated with Veh, N, DT or DTN, **(K)** WM4376 treated with Veh, N, DT or DTN, **(L)** WM4061 treated with Veh, N, DT or DTN, **(M)** WM4200_R treated with Veh, N, DT or DTN. Data represents mean of >6 mice +/- SEM. Significance was determined by two-way ANOVA and Tukey's multiple comparisons tests; significant difference ($P<0.05$) between a growth curve versus another growth curve is indicated by asterisks of the same color as the other growth curve. Data represents mean of $\geq$ 6 mice per treatment group +/- SEM.

Figure S3

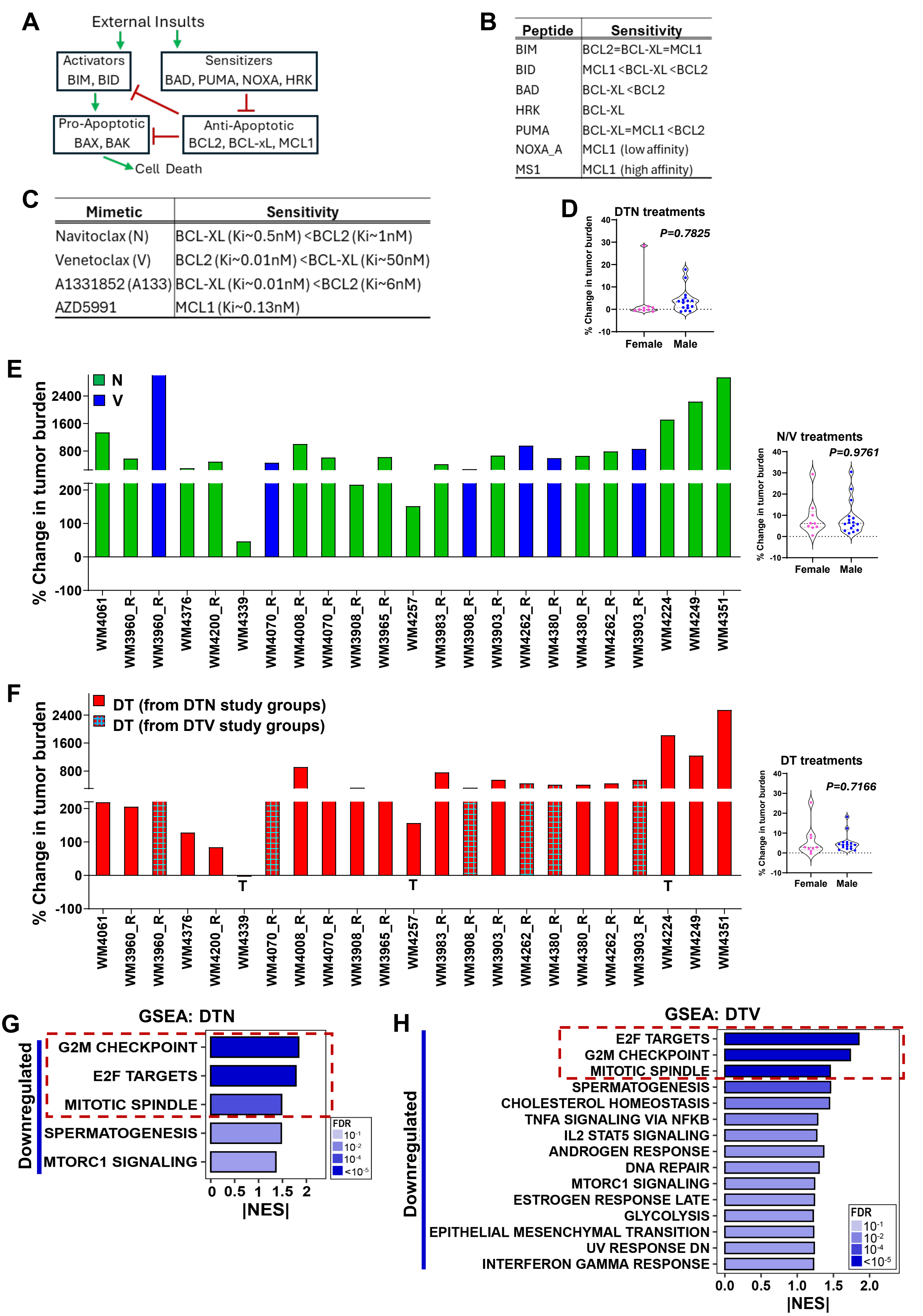
A
External Insults
Activators
BIM, BID
Sensitizers
BAD, PUMA, NOXA, HRK
Pro-Apoptotic
BAX, BAK
Anti-Apoptotic
BCL2, BCL-xL, MCL1
Cell Death
B
Peptide | Sensitivity
BIM | BCL2=BCL-XL=MCL1
BID | MCL1 <BCL-XL <BCL2
BAD | BCL-XL <BCL2
HRK | BCL-XL
PUMA | BCL-XL=MCL1 <BCL2
NOXA_A | MCL1 (low affinity)
MS1 | MCL1 (high affinity)
C
Mimetic | Sensitivity
Navitoclax (N) | BCL-XL (Ki~0.5nM) <BCL2 (Ki~1nM)
Venetoclax (V) | BCL2 (Ki~0.01nM) <BCL-XL (Ki~50nM)
A1331852 (A133) | BCL-XL (Ki~0.01nM) <BCL2 (Ki~6nM)
AZD5991 | MCL1 (Ki~0.13nM)
D
DTN treatments
P=0.7825
% Change in tumor burden
Female
Male
E
N
V
% Change in tumor burden
WM4061, WM3960_R, WM3960_R, WM4376, WM4200_R, WM4339, WM4070_R, WM4008_R, WM4070_R, WM3908_R, WM3965_R, WM4257, WM3983_R, WM3908_R, WM3903_R, WM4262_R, WM4380_R, WM4380_R, WM4262_R, WM3903_R, WM4224, WM4249, WM4351
N/V treatments
P=0.9761
Female
Male
F
DT (from DTN study groups)
DT (from DTV study groups)
% Change in tumor burden
T
DT treatments
P=0.7166
Female
Male
G
GSEA: DTN
Downregulated
G2M CHECKPOINT
E2F TARGETS
MITOTIC SPINDLE
SPERMATOGENESIS
MTORC1 SIGNALING
FDR
|NES|
H
GSEA: DTV
Downregulated
E2F TARGETS
G2M CHECKPOINT
MITOTIC SPINDLE
SPERMATOGENESIS
CHOLESTEROL HOMEOSTASIS
TNFA SIGNALING VIA NFKB
IL2 STAT5 SIGNALING
ANDROGEN RESPONSE
DNA REPAIR
MTORC1 SIGNALING
ESTROGEN RESPONSE LATE
GLYCOLYSIS
EPITHELIAL MESENCHYMAL TRANSITION
UV RESPONSE DN
INTERFERON GAMMA RESPONSE
FDR
|NES|

…continued Figure S3

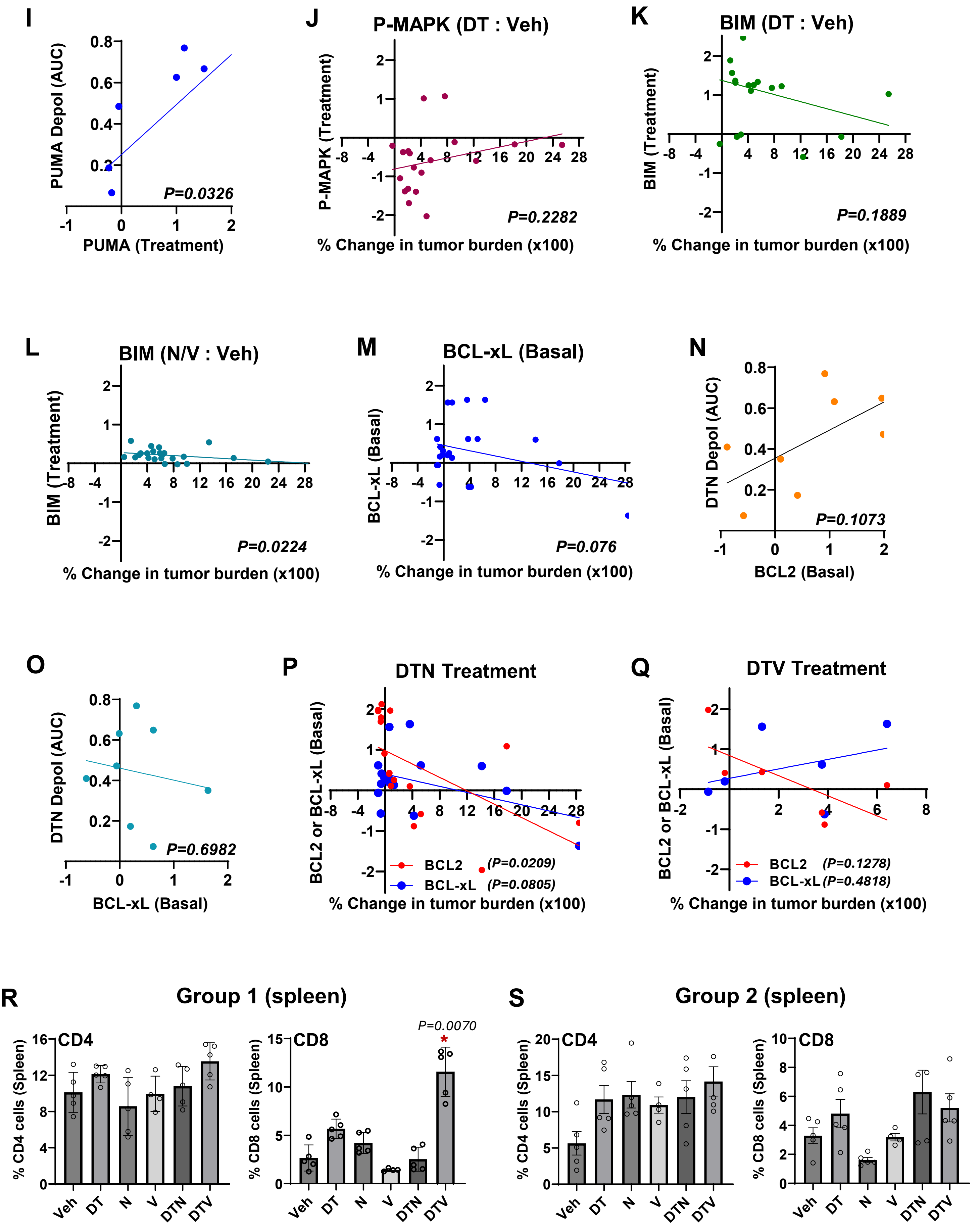

Figure S3. Treatment induced changes in PDX tumor burdens and molecular associations. (A) Schematic figure showing pro- and anti-apoptosis functions of the four types of BCL2 family proteins-BH3 only apoptosis activators, BH3 only apoptosis sensitizers, multidomain pro-apoptosis proteins and multidomain anti-apoptosis proteins. **(B & C)** Tables showing specificity of binding of BH3 peptides *(B)* and BH3 mimetics *(C)* with BH3 only BCL2 family proteins. BCL2 family proteins with similar sensitivities of peptides *(B)* or mimetics *(C)* with the indicated BCL2 family proteins are shown with a "=" , while those with lower sensitivity compared to the one on their left are shown with a "<" . **(D)** Sex disaggregated results of percent changes in tumor burdens of PDXs grown in mice treated with DTN. **(E)** Left panel shows column graph with percentage changes in PDX tumor burdens from the start to end or maximum ~30 days of treatment with N (Navitoclax) or V (Venetoclax). Green bars represent N, and blue bars represent V treatments. Right panel shows sex-disaggregated changes in tumor burdens following treatments. **(F)** Left panel shows column graph with percentage changes in PDX tumor burdens from start to end or maximum ~30 days of treatment with DT or T. Solid red bars represent treatments in mice of the DTN treatment groups, while hashed red bars represent treatments from the DTV treatment groups. The T only treatments are indicated below the respective columns. Right panel shows sex-disaggregated changes in tumor burdens following treatments. **(G & H)** GSEA analysis of the most significantly altered transcriptional networks in early harvest tumors from mice treated with DTN *(G)* and DTV *(H)*, compared to vehicle. The three most significant transcriptional networks are indicated inside red dashed rectangles. **(I)** Linear regression analysis of the association of DTN induced PUMA levels in eight PDXs with mitochondrial membrane depolarizations induced by PUMA peptide. Significance determined by best-fit analysis. **(J-L)** Linear regression analysis of the association of DT/T-treatment induced P-MAPK *(J)* and BIM *(K)* proteins with percent changes in DT treatment-altered tumor burdens, and N/V treatment-induced BIM with percent changes in N/V treatment-altered tumor burdens *(L)*. Significance derived from best-fit analysis and *P*-values indicated at the bottom right of each graph. **(M)** Linear regression analysis of the association of basal BCL-xL protein with percent change in PDX tumor burdens following treatments with DTN/V. Significance derived from best-fit analysis. **(N & O)** Linear regression analysis of the association of BCL2 *(N)* and BCL-xL *(O)* protein levels with mitochondrial membrane depolarizations induced by DTN combination treatment in eight PDX models. Significance derived from best-fit analysis and *P*-values indicated at the bottom right corner of each graph. **(P & Q)** Linear regression analysis of the association of basal BCL2 (red) or BCL-xL (blue) protein levels with DTN-induced *(P)* or DTV-induced *(Q)* changes in PDX tumor burdens. **(R & S)** IHC analysis of splenic CD4 and CD8 T cells shown as percentage of total cells in the spleens of CD34+ humanized mice bearing sub-cutaneous WM4380_R tumors and treated daily with the indicated therapeutic agents in two groups. Data represents mean of ten random 1 mm^2 measurements per treatment +/- SD. Significance determined by one-way ANOVA and Kruskal-Wallis multiple comparisons testing and significant *P-values* indicated by asterisks.

Figure S4

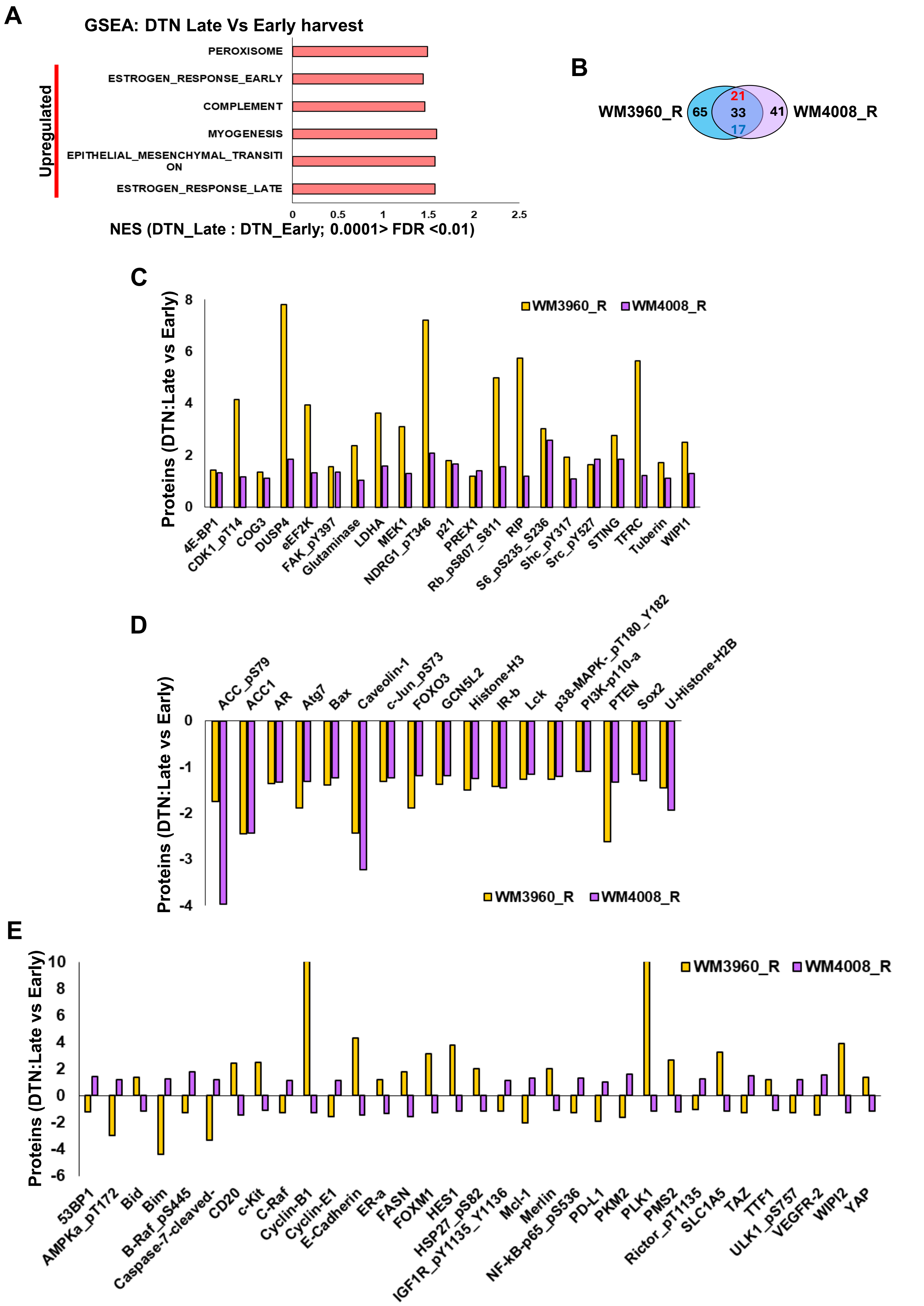
A
GSEA: DTN Late Vs Early harvest
Upregulated
PEROXISOME
ESTROGEN_RESPONSE_EARLY
COMPLEMENT
MYOGENESIS
EPITHELIAL_MESENCHYMAL_TRANSITION
ESTROGEN_RESPONSE_LATE
0 0.5 1 1.5 2 2.5
NES (DTN_Late : DTN_Early; 0.0001> FDR <0.01)
B
WM3960_R 65 21 33 17 41 WM4008_R
C
Proteins (DTN:Late vs Early)
WM3960_R WM4008_R
4E-BP1 CDK1_pT14 COG3 DUSP4 eEF2K FAK_pY397 Glutaminase LDHA MEK1 NDRG1_pT346 p21 PREX1 Rb_pS807_S811 RIP S6_pS235_S236 Shc_pY317 Src_pY527 STING TFRC Tuberin WIPI1
D
Proteins (DTN:Late vs Early)
ACC_pS79 ACC1 AR Atg7 Bax Caveolin-1 c-Jun_pS73 FOXO3 GCN5L2 Histone-H3 IR-b Lck p38-MAPK-_pT180_Y182 PI3K-p110-a PTEN Sox2 U-Histone-H2B
WM3960_R WM4008_R
E
Proteins (DTN:Late vs Early)
WM3960_R WM4008_R
53BP1 AMPKa_pT172 Bid Bim B-Raf_pS445 Caspase-7-cleaved- CD20 c-Kit C-Raf Cyclin-B1 Cyclin-E1 E-Cadherin ER-a FASN FOXM1 HES1 HSP27_pS82 IGF1R_pY1135_Y1136 Mcl-1 Merlin NF-kB-p65_pS536 PD-L1 PKM2 PLK1 PMS2 Rictor_pT1135 SLC1A5 TAZ TTF1 ULK1_pS757 VEGFR-2 WIPI2 YAP

F

Fig 4A (WM3960_R)	
**Tukey's multiple comparisons test**	**P Value**
Veh vs. DT	<0.0001
Veh vs. DTN	<0.0001
Veh vs. N	0.1075
DT vs. DTN	0.0003
DT vs. N	0.0004
DTN vs. N	<0.0001

G

Fig 4B (WM4008_R)	
**Tukey's multiple comparisons test**	**P Value**
Veh vs. DT	0.8816
Veh vs. DTN	<0.0001
Veh vs. N	0.8856
DT vs. DTN	<0.0001
DT vs. N	>0.9999
DTN vs. N	<0.0001

Figure S4. Molecular associations of treatment responses. (A) GSEA analysis of the significantly altered (FDR>0, <0.01) transcriptional networks in RNA of late harvest versus early harvest tumors from DTN treated mice. **(B)** Venn diagram of RPPA analyzed proteins showing similarly upregulated (red font), similarly downregulated (blue font), or differentially regulated (black font) proteins in WM3960_R (yellow circle) and WM4008_R (purple circle) late harvest tumors harvested from DTN treated mice. **(C–E)** Bar graphs showing the expression of the above-identified similarly upregulated *(C),* similarly downregulated *(D)*, or differentially regulated *(E)* proteins in the late resistant tumors of WM3960_R (yellow) and WM4008_R (purple). **(F-G)** P-values of treatment induced alterations between tumor growth curves from Fig 4A *(F)* and 4B *(G).*

Figure S5

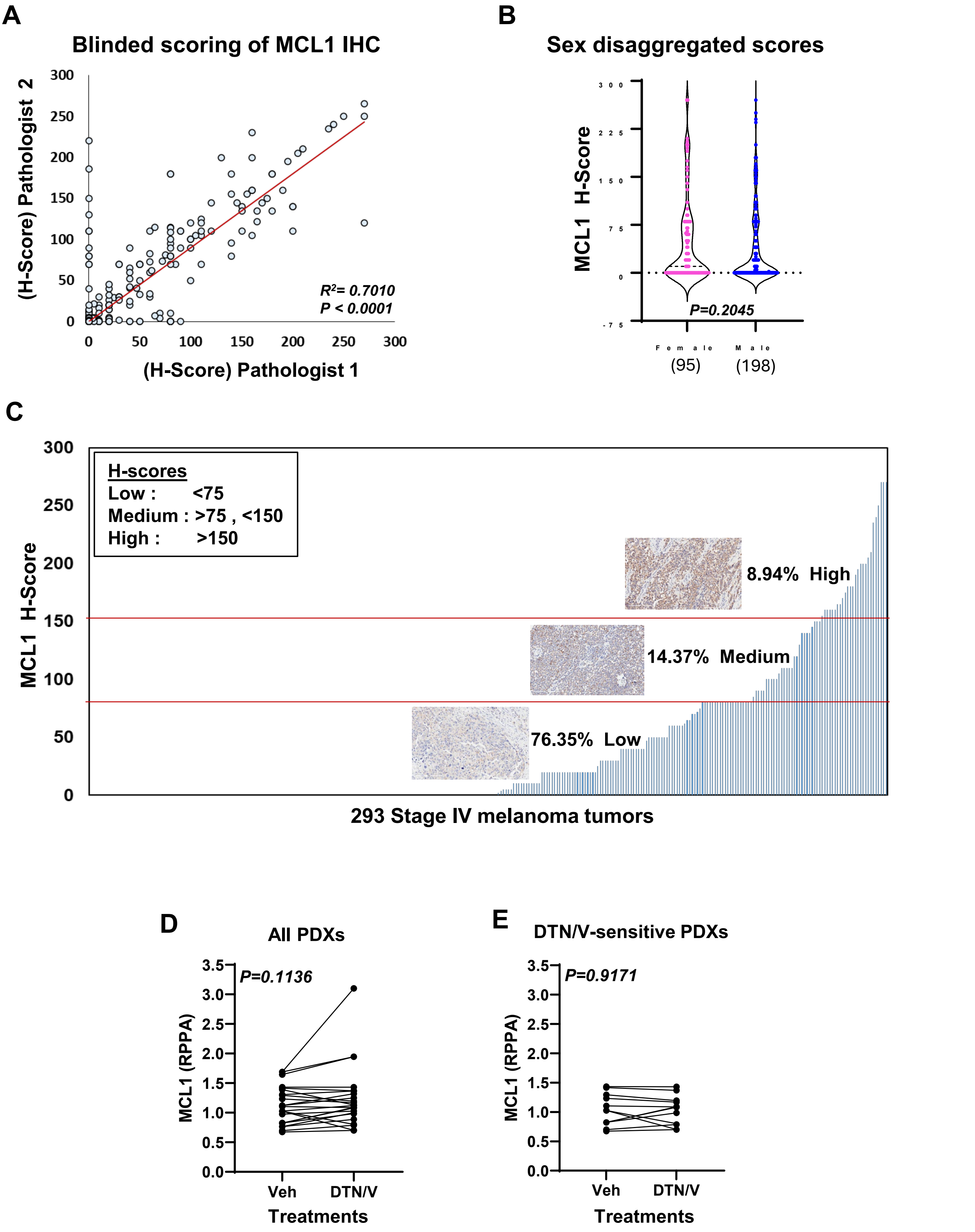
A
Blinded scoring of MCL1 IHC
(H-Score) Pathologist 2
(H-Score) Pathologist 1
R²= 0.7010
P < 0.0001
B
Sex disaggregated scores
MCL1 H-Score
P=0.2045
Female
Male
(95)
(198)
C
H-scores
Low : <75
Medium : >75 , <150
High : >150
MCL1 H-Score
8.94% High
14.37% Medium
76.35% Low
293 Stage IV melanoma tumors
D
All PDXs
P=0.1136
MCL1 (RPPA)
Veh
DTN/V
Treatments
E
DTN/V-sensitive PDXs
P=0.9171
MCL1 (RPPA)
Veh
DTN/V
Treatments

Figure S5. MCL1 expression in human melanoma. (A) IHC staining with MCL1 antibody from Cell Signaling Technologies (clone D5V5L) validated in-house in 293 tumors from stage IV melanoma patients. H-scoring of staining intensities was performed independently by two pathologists in a blinded study, and linear regression analysis to validate concordance of MCL1 quantification. **(B)** Sex-disaggregated MCL1 H-scores in the above patient samples. Significance determined by two-sided t-test. **(C)** Stratification of the above melanoma patient tumors into three different MCL1 expression groups based on H-score intensities. **(D & E)** MCL1 protein levels in Veh and DTN/V treated tumors of all PDXs *(D)* and DTN/V-sensitive PDXs only *(E)*; and significance determined by two-sided t-tests.

Figure S6

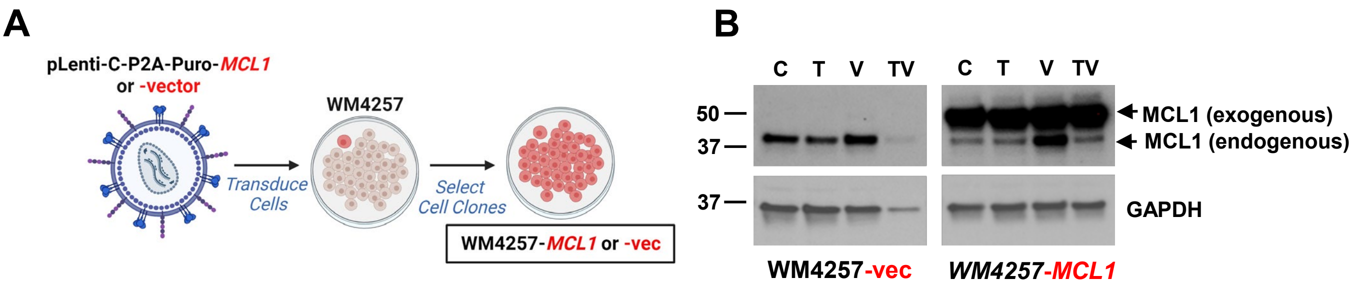

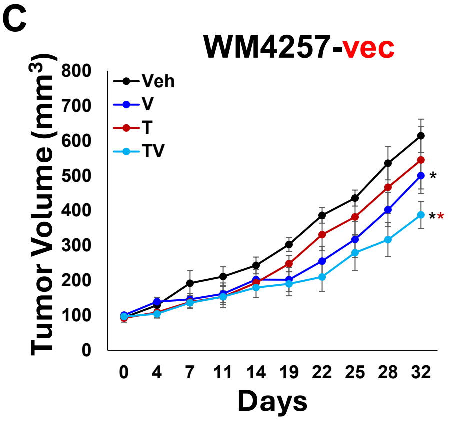

Comparisons	P-values
Veh vs V; T; TV	0.0096; 0.1067; 0.0002
T vs V; TV	0.7896; 0.6112
TV vs V	0.1446

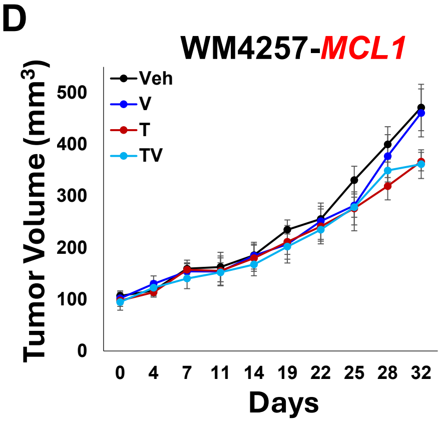

Comparisons	P-values
Veh vs V; T; TV	0.0721; 0.0624; 0.2794
T vs V; TV	>0.9999; 0.8182
TV vs V	0.8346

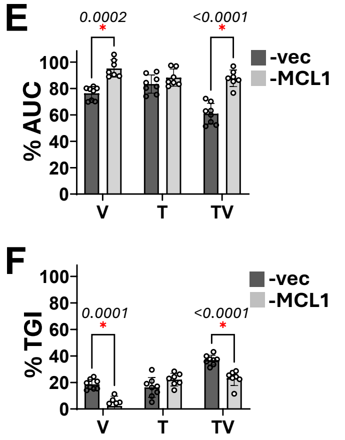

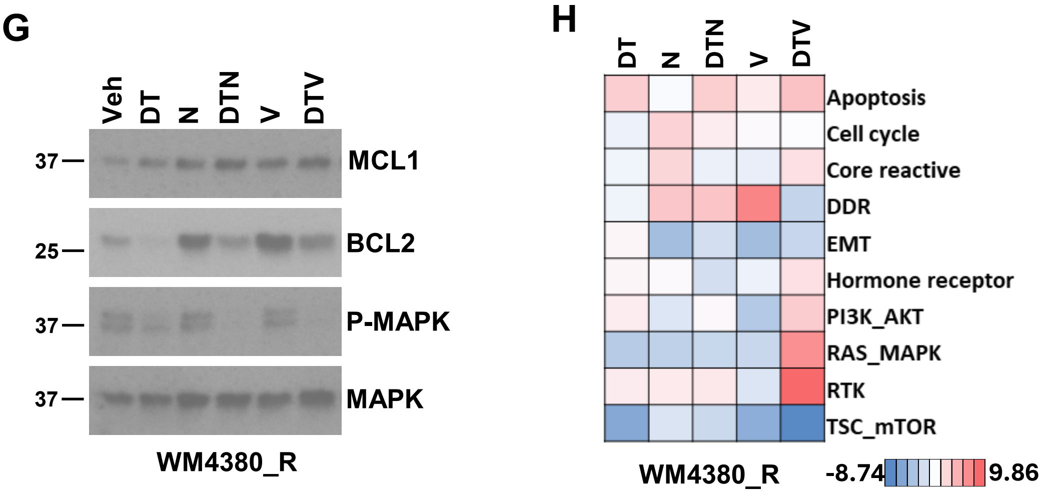

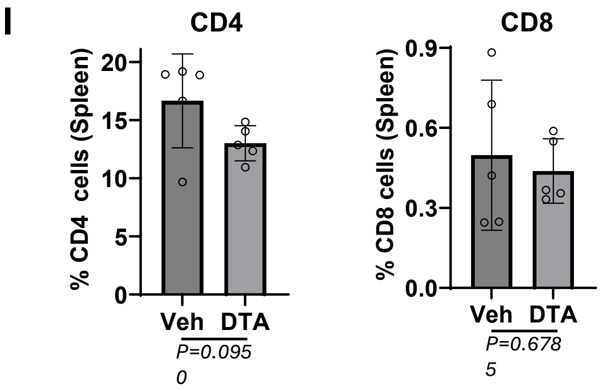

Figure S6. Role of MCL1 in resistance to BCL2 inhibition. (A) Generation of MCL1-overexpressing clones or its vector control (WM4257_R-*MCL1* or -*vec*) in WM4257_R PDX-derived cells; Created in BioRender. Loftin, K. (2026) https://BioRender.com/or3m87t. **(B)** Western blotting to assess the expression of exogenous and endogenous MCL1 protein in cells after treatment with the indicated drugs**.** GAPDH protein was used as loading control. Molecular weight markers in kDa are indicated on the left of each blot. Results are from two separate gels processed in parallel and data represents reproducible results from two separate experiments. **(C &D)** Tumor growth of WM4257_R-*vec (C)* and WM4257_R-*MCL1 (D)* in mice treated daily with vehicle (Veh), 75mg/kg Venetoclax (V), 1mg/kg Trametinib (T) or their combination, TV. Data is from > 6 mice per group +/- SEM; significance determined by two-way ANOVA and Tukey's post-hoc tests; significant difference *(P<0.05)* of any growth curve versus others is indicated by asterisks of the same color as the other growth curves and *P*-values are shown below the graphs. **(E)** Bar graphs of Area Under the Curve (AUC) analysis showing percent changes of treatments versus vehicle. Significance (*P<0.05*) by t-tests indicated with asterisks. (**F**) Tumor Growth Inhibition (TGI) shown as percent changes versus vehicle. Significance (*P<0.05*) by t-tests indicated with asterisks. **(G)** Western blots of indicated proteins in WM4380_R tumor lysates from the tumor growth study in Figure S2E. MAPK (ERK2) was used as loading control. Molecular weight markers in kDa are indicated on the left of each blot. Results are from one experiment and two separate gels processed in parallel. Data represents results from two independent experiments. **(H)** Differentially regulated protein signaling networks from RPPA analysis of WM4380_R tumor lysates from the tumor growth study in Figure S2E. **(I)** IHC analysis of splenic CD4 and CD8 T cells shown as percentage of total cells in the spleens of CD34+ humanized mice bearing sub-cutaneous WM4380_R tumors and treated daily with vehicle or DTA. Data represents mean of five random 1 mm^2 measurements +/- SD in single mice, and significance determined by two-sided t-tests and *P*-values indicated below graph.

Figure S7

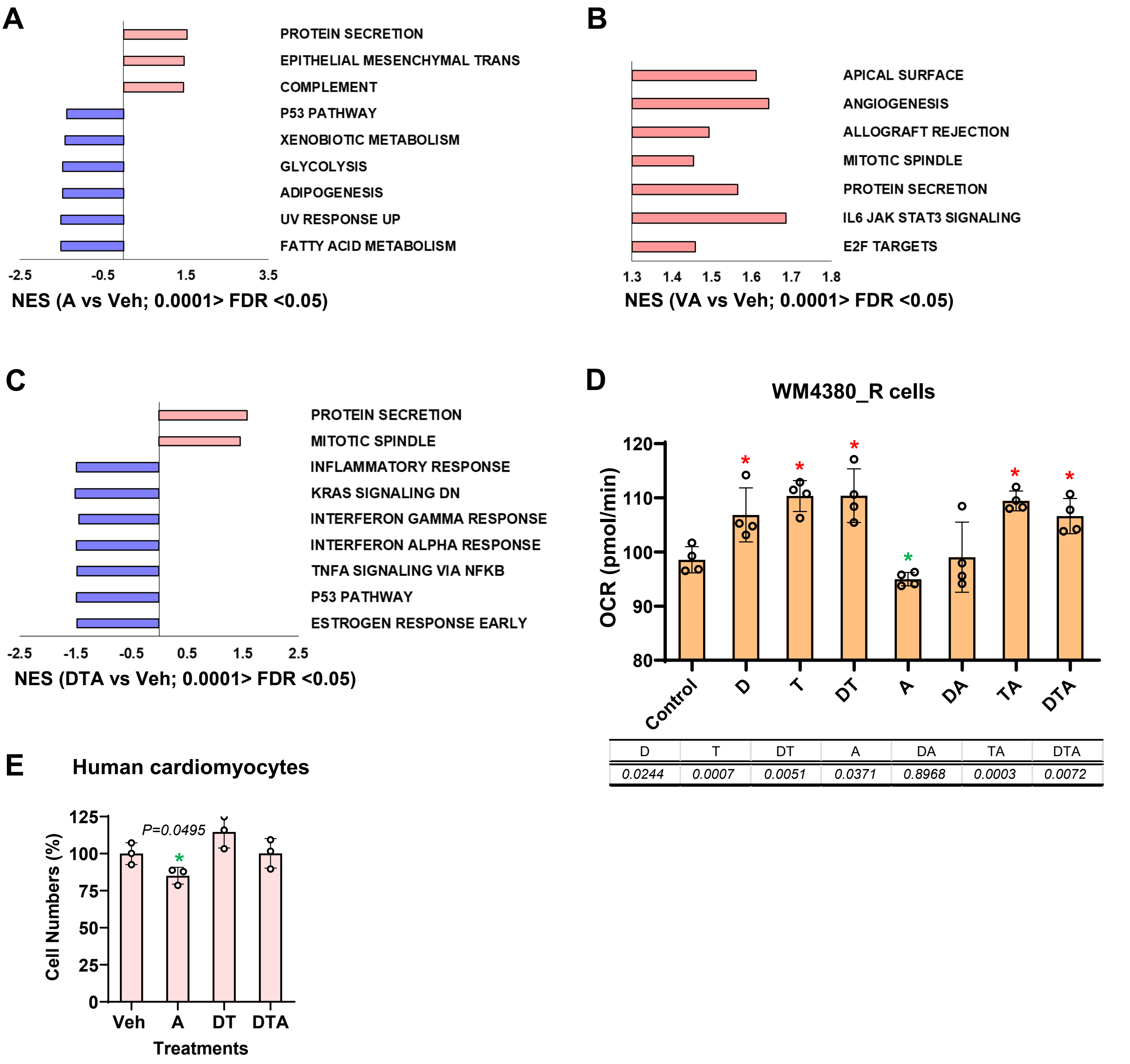

D	T	DT	A	DA	TA	DTA
0.0244	*0.0007*	*0.0051*	*0.0371*	*0.8968*	*0.0003*	*0.0072*

Figure S7. Effect of MCL1 and MAPK inhibition on human melanoma PDXs, cells and cardiomyocytes. (A-C) GSEA analysis showing significantly altered (FDR>0, <0.05) transcriptional networks in RNA of WM4380_R tumors after treatment with A *(A)*, VA *(B)*, or DTA *(C)*. **(D)** Basal oxygen consumption rate (OCR) in WM4380_R PDX derived cells after 24h treatment with the indicated inhibitors, 0.1μM D, 0.03μM T, 1μM A or their combinations. Data represents biological quadruplicates +/- SD; significance determined by two-sided t-tests and colored asterisks indicate significantly higher (red) or significantly lower (green) OCR versus vehicle, with *P*-values shown below graph. **(E)** Effect of A, DT and DTA treatments on the viability of human cardiomyocytes, measured as cell numbers after 72 h of treatments. Two-sided t-tests were used to assess significance *(P<0.05)* and indicated on the graph.
