# Supplementary material for: Personalized targeting of BCL2 family proteins overcomes acquired resistance to BRAF-MEK inhibitors in preclinical melanoma
Source: Nat Commun. 2026 Jun 26;17:7582. doi: 10.1038/s41467-026-74691-9 (PMC13421458; doi:10.1038/s41467-026-74691-9)
Supplement: Supplementary file 3 — Reporting Summary [file 41467_2026_74691_MOESM3_ESM.pdf]

nature portfolio

Corresponding author(s): Y.N. Vashisht Gopal

Last updated by author(s): Apr 17, 2026

Reporting Summary

Nature Portfolio wishes to improve the reproducibility of the work that we publish. This form provides structure for consistency and transparency in reporting. For further information on Nature Portfolio policies, see our Editorial Policies and the Editorial Policy Checklist.

Statistics

For all statistical analyses, confirm that the following items are present in the figure legend, table legend, main text, or Methods section.

n/a	Confirmed	
☐	☒	The exact sample size (*n*) for each experimental group/condition, given as a discrete number and unit of measurement
☐	☒	A statement on whether measurements were taken from distinct samples or whether the same sample was measured repeatedly
☐	☒	The statistical test(s) used AND whether they are one- or two-sided<br>*Only common tests should be described solely by name; describe more complex techniques in the Methods section.*
☐	☒	A description of all covariates tested
☐	☒	A description of any assumptions or corrections, such as tests of normality and adjustment for multiple comparisons
☐	☒	A full description of the statistical parameters including central tendency (e.g. means) or other basic estimates (e.g. regression coefficient) AND variation (e.g. standard deviation) or associated estimates of uncertainty (e.g. confidence intervals)
☐	☒	For null hypothesis testing, the test statistic (e.g. F, t, r) with confidence intervals, effect sizes, degrees of freedom and P value noted<br>*Give P values as exact values whenever suitable.*
☒	☐	For Bayesian analysis, information on the choice of priors and Markov chain Monte Carlo settings
☒	☐	For hierarchical and complex designs, identification of the appropriate level for tests and full reporting of outcomes
☐	☒	Estimates of effect sizes (e.g. Cohen's *d*, Pearson's *r*), indicating how they were calculated

Our web collection on statistics for biologists contains articles on many of the points above.

Software and code

Policy information about availability of computer code

Data collection	Bioenergetics Stress tests and OCR analysis- Agilent Seahorse<br>Scanning of IHC slides - Aperio Scanscope (Aperio/Leica Microsystems)<br>Electrophysiology studies - Maestro-361 (Axion Biosystems)
Data analysis	Graphpad Prizm - Statistical analysis and graphical presentations.<br>Microsoft Excel - Statistical analysis and graphical presentations.<br>Gene Set Enrichment Analysis (GSEA)- Open source (https://www.gsea-msigdb.org)<br>Clustering of signaling networks - GeneCluster 3.0<br>Generation of heatmaps - Treeview<br>Batch corrections for RPPA data using MBatch - Open Source (https://bioinformatics.mdanderson.org/public-software/mbatch/).<br>Electrophysiology studies - AxIS Suite 3.12<br>IHC analysis - Aperio ImageScope

For manuscripts utilizing custom algorithms or software that are central to the research but not yet described in published literature, software must be made available to editors and reviewers. We strongly encourage code deposition in a community repository (e.g. GitHub). See the Nature Portfolio guidelines for submitting code & software for further information.

Data

Policy information about availability of data

All manuscripts must include a data availability statement. This statement should provide the following information, where applicable:

- Accession codes, unique identifiers, or web links for publicly available datasets
- A description of any restrictions on data availability
- For clinical datasets or third party data, please ensure that the statement adheres to our policy

Deidentified RNAseq and DNA mutation data of melanoma PDXs in the study were submitted to NCBI GEO (accession number GSE298507). Reverse Phase Protein Array data of PDXs is included in the supplementary data. Additional data are available from the authors upon reasonable request.

Research involving human participants, their data, or biological material

Policy information about studies with human participants or human data. See also policy information about sex, gender (identity/presentation), and sexual orientation and race, ethnicity and racism.

Reporting on sex and gender	Sex of patients from whom the patient-derived samples were generated is included in Figure S1I and S5B.
Reporting on race, ethnicity, or other socially relevant groupings	This study is focused on non-acral cutaneous melanoma, which almost always affects white individuals, and the patient samples in the study are also from the same racial group.
Population characteristics	Older age over 50 years is a predominant characteristic of cutaneous melanoma patients, while there are some instances of early age. Our patient derived samples represent a similar distribution of age of patients.
Recruitment	Patient samples were recruited blindly based on availability and molecular characteristics that would allow unbiased testing of therapeutics used in this study.
Ethics oversight	M.D. Anderson Cancer center Institutional Review Board.

Note that full information on the approval of the study protocol must also be provided in the manuscript.

Field-specific reporting

Please select the one below that is the best fit for your research. If you are not sure, read the appropriate sections before making your selection.

☒ Life sciences ☐ Behavioural & social sciences ☐ Ecological, evolutionary & environmental sciences

For a reference copy of the document with all sections, see nature.com/documents/nr-reporting-summary-flat.pdf

Life sciences study design

All studies must disclose on these points even when the disclosure is negative.

Sample size	For animal experiments, sample sizes were determined based on power calculations with a type I error < 0.05 to detect significant differences. No statistical method was used to predetermine sample size for other experiments. Sample sizes were determined based on determination of significance and reproducibility.
Data exclusions	No data were excluded.
Replication	The number of biological independent samples is reported in the figure legends.
Randomization	Mice implanted with PDX tumors were blindly randomized prior to initiation of therapeutic treatment studies. Tumor sample collection from mice at specific times was also performed randomly.
Blinding	Research technician was provided the therapeutic treatments labeled as alphabets or numbers rather than the names of the treatments prior to administering them to mice.

Reporting for specific materials, systems and methods

We require information from authors about some types of materials, experimental systems and methods used in many studies. Here, indicate whether each material, system or method listed is relevant to your study. If you are not sure if a list item applies to your research, read the appropriate section before selecting a response.

Materials & experimental systems

n/a	Involved in the study
	☒ Antibodies
	☒ Eukaryotic cell lines
☒	☐ Palaeontology and archaeology
	☒ Animals and other organisms
	☒ Clinical data
☒	☐ Dual use research of concern
☒	☐ Plants

Methods

n/a	Involved in the study
☒	☐ ChIP-seq
☒	☐ Flow cytometry
☒	☐ MRI-based neuroimaging

Antibodies

Antibodies used	anti-human BCL2, MAPK (ERK2), Phospho-MAPK (ERK1/2), MCL1, GAPDH at 1:1000 dilutions for western blotting. Antibodies used for RPPA are listed on the RPPA core facility website- https://www.mdanderson.org/research/research-resources/core-facilities/functional-proteomics-rppa-core.html
Validation	Antibodies for western blotting were purchased from Cell Signaling Technologies, which provides a validation statement and lot numbers. We further make sure that we are detecting the correct protein by assessing molecular weight of detected proteins using molecular weight markers. Antibodies used for RPPA are validated by the RPPA core facility website.

Eukaryotic cell lines

Policy information about cell lines and Sex and Gender in Research

Cell line source(s)	Singel cell suspensions were generated from PDXs used in the study, which are sourced from the Wistar Institute and publicly available. Gender for each is indicated in the results in Figure 2B.
Authentication	Cell lines were authenticated using STR fingerprinting.
Mycoplasma contamination	All cells used in the study were found negative for mycoplasma.
Commonly misidentified lines (See ICLAC register)	None in this study.

Animals and other research organisms

Policy information about studies involving animals; ARRIVE guidelines recommended for reporting animal research, and Sex and Gender in Research

Laboratory animals	NSG mice and NSG-SGM3 (NOD.Cg-Prkdc Il2rg/SzJ) mice age ~8 weeks at the beginning of experiments
Wild animals	None
Reporting on sex	Both male and female mice were used in the study, and analyis included sex as a variable.
Field-collected samples	None
Ethics oversight	Obtained M.D. Anderson Institutional Animal Care and Use Committee (IACUC) approval for all animal studies.

Note that full information on the approval of the study protocol must also be provided in the manuscript.

Clinical data

Policy information about clinical studies

All manuscripts should comply with the ICMJE guidelines for publication of clinical research and a completed CONSORT checklist must be included with all submissions.

Clinical trial registration	No clinical trial
Study protocol	The clinical samples were retrospective and obtained from our MDACC MelCORE repository under an IRB approved use protocol.
Data collection	Samples were obtained as formalin fixed slides.
Outcomes	Outcomes are not reported in this study.

Plants

Seed stocks	NA
Novel plant genotypes	NA
Authentication	NA
