# Supplementary material for: Personalized targeting of BCL2 family proteins overcomes acquired resistance to BRAF-MEK inhibitors in preclinical melanoma
Source: Nat Commun. 2026 Jun 26;17:7582. doi: 10.1038/s41467-026-74691-9 (PMC13421458; doi:10.1038/s41467-026-74691-9)
Supplement: Supplementary file 2 — Peer Review file [file 41467_2026_74691_MOESM2_ESM.pdf]

nature portfolio

Peer Review File

Personalized targeting of BCL2 family proteins overcomes acquired resistance to BRAF-MEK inhibitors in preclinical melanoma

Corresponding Author: Dr Vashisht YN

This file contains all reviewer reports in order by version, followed by all author rebuttals in order by version.

Version 1:

Reviewer comments:

Reviewer #1

(Remarks to the Author)
This manuscript presents a number of interesting results but lacks structure; the data is a bit scrambled.
This doesn´t help the fact that, as the authors point out, the role of Bcl-proteins in melanoma response to MAPKi has previously been published. Thus, there is a lack of novelty.
There are some fundamental flaws when explaining some of the results. The manuscript should be edited to be more concise, as it is sometimes difficult to follow the thread. This is exemplified in page 3 of the Results section, when describing the data in Figure 3B.
There is quite some data that doesn´t add much to the main point of the study but that occupies significant proportions of the text. For instance, the bottom 2 paragraphs in page 5 of the Results section could be summarize in a couple of sentences within the discussion section.
The most novel data is found in figure 4C that yet it gets a bit lost among so many less rel3evant and novel results. For example, data in figures 4E-H do not add anything meaningful to the importance of MCL-1 in melanoma response to MAPKi. Since Mcl-1 had been part of the story form the beginning, why not directly assess its expression in recurrent tumors to introduce the following results overexpressing and/or inhibiting Mcl-1. Again, it shows a lack of focus that doesn´t help the reader to capture the positives of this study.
Similarly, the data presented in Figure 7 regarding cardiotoxicity seems more like the beginning of a new story rather than the end of this one.
In summary, the main issue with this manuscript is that almost every figure introduces a new variable, potential mechanism of action or the involvement of a certain pathway. As a result, the manuscripts fails to convincingly address any single question.
This study requires some rethinking, structuring to then rewrite a more round, focused story.
For starters, I would suggest focusing the study/results on BRAF mutant PDXs.

Reviewer #2

(Remarks to the Author)
This study examines the effect of inhibiting anti-apoptotic proteins in combination with MAPKi in PDX models of melanoma. Effects range from complete tumor growth inhibition to no effect. The authors make good effort in identifying pathways using protein arrays but these experiments do not yield a clear biomarker. The following concerns arise:
1. Navitoclax has been abandoned from clinical development. What is the rational to use it in these experiments?
2. What is the effect of the combination on immune cells? Immune PDX models have been developed so it is unclear why these are not used.
3. How is BCL2/XL regulated by MAPKI and why is it of use to wait for resistance development before adding the anti-apoptotic protein inhibitors?

Reviewer #3

(Remarks to the Author)
The manuscript by Vashisht Gopal and colleagues studies the impact of BCL2 inhibition on BRAFi/MEKi treatments in a large collection of molecularly characterized melanoma PDXs and patient samples. The findings indicated that increased

BCL-2 protein levels are associated with treatment resistance. Single BCL2 inhibitors (BCL2i) such as Navitoclax or Venetoclax were ineffective against BRAFi+MEKi-resistant PDXs. However, combining BRAFi+MEKi with BCL2i induced potent tumor inhibition and regression, correlated with high basal BCL2 and low basal MCL-1 levels. In contrast, high basal MCL-1 and treatment-induced MCL1 were associated with resistance to BRAFi+MEKi+Venetoclax and BRAFi+MEKi+Navitoclax. Additionally, MEKi treatment counteracted mitochondrial OxPhos inhibition and cardiac troponin activation caused by MCL1i.

The manuscript is well organized. The methods are appropriate, and the results are clearly described and properly discussed. References are relevant and quite updated, but a few references are missing. Thus, this study confirms previous articles exploring the potential use of BH3 mimetics (such as venetoclax, navitoclax and AZD5991) to enhance DT treatment. We have some comments and suggestions that would benefit the manuscript.
Some detailed comments can be found below:

• Conclusions are not new, every PDX/patient responds slightly differently. The authors do not mention the need for biomarkers for personalized medicine.
• That the anti-apoptotic BCL-2 family proteins mediate resistance to BRAFi+MEKi in melanoma is not new and has been previously reported by several groups. Please include the appropriate references in the manuscript such as PMID: 31727888, PMID: 31727958, PMID: 37460459.
• The authors should provide evidence on BCL-xL inhibition, that has not been explored in the manuscript.
• Is the increase of BIM expression a biomarker for BCL2 inhibition? It has been reported that MAPK inhibition leads to BIM accumulation.
• It would be interesting if the authors explored the potential patients stratification using BCL2 and BCL-xL protein expression, not only MCL-1.
• In figure 5E the authors should represent MCL-1 increased expression individually, since there is only one clear PDX sample that doubles the expression of this protein.
• Have the authors checked in figure 6E if the tumor treated with DT adapts through MCL-1?
• BCL family of proteins should be changed to BCL-2 family of proteins throughout the text
• The authors should explore the expression of sensitizer BCL-2 family proteins such as NOXA, BAD and HRK, that directly affect anti-apoptotic proteins pro-survival efficacy.

Minor comments:
• Please revise the manuscript and properly reference the figures. Some figures are not well indicated in the text

Reviewer #4

(Remarks to the Author)
The manuscript by Gopal and colleagues reports that combining inhibition of BCL2 family members can increase the response of BRAFV600E-mutant melanoma response to BRAF and MEK inhibition. While a prior phase II clinical trial failed to show any significant improvement of combining navitoclax with BRAF and MEK inhibition, the authors sought to see whether stratifying patient-derived melanoma samples could reveal an impact. Their findings indicate that patients with high levels of BCL2 did show a therapeutic benefit when either venetoclax (V) or navitoclax (N) were added to dabrafenib (D) and trametinib (T). In some samples, they found that increases in MCL1 expression facilitated resistance. Therefore, they showed using MCL1 overexpression or MCL1 inhibition could render PDX samples resistant or susceptible to D and T respectively. Finally, they argued that MCL1 inhibition, which has been linked to cardiotoxicity in phase I clinical trials, did result in a signaling associated with repression of oxidative phosphorylation and troponin release, but this appeared to be repressed by D and T treatment.
The findings of this study could be therapeutically beneficial in a devastating disease; therefore, the study is of interest. Overall, the study was well performed with rigorous experimentation, data analyses, and robust methodologies including a large panel of PDX samples. Despite these strengths, some weaknesses remain that should be addressed.
1. The manuscript seems to have added the impact on cardiomyocytes to the story at a later date. No methods are provided for any of these experiments. How much of the drugs are being used? What is the source of the human cardiomyocytes? How is troponin IHC being assessed?
2. How can the authors disentangle the impact of combination therapies inducing death versus impacting oxidative phosphorylation in Figure 7? If the impact on oxidative phosphorylation is merely due to the early stages of BAX/BAK-triggered mitochondrial permeabilization (so called MOMP), these data may be misinterpreted.
3. The response of cancer cells to BCL2 antagonists is often complex and not well predicted by expression alone. In this study, the authors do assess protein levels, which are more reflective of expression than mere mRNA expression. However, even with this it has been a struggle to predict which tumors respond. Thus, many laboratories use a functional assay to predict responses, called BH3 profiling (PMID: 33217342, 34315868, 37210412, etc,). This approach has the benefit of assessing not only the upregulation of anti-apoptotic family members, but also the repression of pro-apoptotic family members. Thus, it might provide a more comprehensive sense of which melanoma samples might be predicted to respond. Also, it might explain why some tumors do not upregulate MCL1 expression, but become resistant to DTV or DTN. Perhaps these samples downmodulate pro-apoptotic molecules. Thus, the overall functional assessment would be that they are less primed to MCL1 inhibitors or peptides.

4. A significant portion of the manuscript are linked to impact on gene expression (S4, S7, Figure 7, etc.). While I understand that this allows signatures to be assessed such as impact of oxidative phosphorylation, mTOR, etc. none of these are validated, nor is there any real interpretation of the data. This seems like a missed opportunity.

Reviewer #5

(Remarks to the Author)
I co-reviewed this manuscript with one of the reviewers who provided the listed reports. This is part of the Nature Communications initiative to facilitate training in peer review and to provide appropriate recognition for Early Career Researchers who co-review manuscripts.

Version 2:

Reviewer comments:

Reviewer #1

(Remarks to the Author)
The manuscript has gained some clarity but it is still complicated to follow. The data doesn´t build up into sound, consistent story. Throughout the text, there are too many speculative statements. This is mainly because there is too much variability in tumor response, gene expression correlations etc… and one cannot draw general conclusions. I still find it doesn´t add enough novelty to the field.
- 145 Navitoclax or Venetoclax induced minimal to no growth inhibition of all PDXs in the study. This is just not true, there 6-7 cases where N o V produced obvious anti-tumor effects.
- Lanes 153-165. This paragraph is filled with speculative conclusions that are not sufficiently substantiated by the data
- Figure 3B. It is really difficult to withdraw such conclusions from the heatmap thereby presented.
- 187 melanoma cells derived from eight PDXs showed a similar correlation of basal BCL2 levels with DTN treatment-induced depolarizations (Fig S3D) but not with BCL-xL levels (Fig S3E), This correlation is not statistically significant
- Data in figure 3G-H, is not solid enough to draw such definitive conclusions.
The differences between WM3690_R-vec and WM3690_R-MCL1 tumors responses to treatments look weak. "Most importantly, compared to -vec tumors (Fig 5I and Fig S6C), -MCL1 tumors were significantly resistant to growth inhibition by single agent N or V or DTN/DTV combination treatments (Fig 5J and S6D), confirming the role of MCL1 in resistance. This sentence is not sustained by the data presented.
- As suggested in my previous revision, the addition of data on cardiac toxicity doesn´t increase the novelty of the study.

Reviewer #3

(Remarks to the Author)
The authors answered all our comments and the manuscript is now acceptable for publication

Reviewer #4

(Remarks to the Author)
The revised submission by Gopal and colleagues largely responds to my prior critiques satisfactorily. Most importantly, the revised manuscript provides a revised methods section to explain how the authors used cultured human cardiomyocytes. While these experiments are important, the authors must provide the limitation in the manuscript about the use of IPS-derived cardiomyocyte cultures. Such cultured IPS-derived and differentiated cardiomyocytes are typically most representative of embryonic (that is immature) cardiomyocytes. As a result, these cultured IPS-derived cardiomyocytes typically feature more embryonic metabolism including the utilization of glucose to support oxidative phosphorylation. In contrast, adult cardiomyocytes alter their metabolism to primarily utilize fatty acids to support oxidative phosphorylation. While the authors' studies are not necessarily impacted by this model, it has been reported that MCL1 functions to promote fatty acid oxidation (see Wright et al. Mol Cell 2024). Therefore, models (such as this one) that utilize embryonic cardiomyocyte cultures may not faithfully reflect the toxicities associated with MCL1 inhibition. This limitation should be pointed out in the discussion.

Reviewer #5

(Remarks to the Author)
I co-reviewed this manuscript with one of the reviewers who provided the listed reports. This is part of the Nature Communications initiative to facilitate training in peer review and to provide appropriate recognition for Early Career Researchers who co-review manuscripts.

Version 3:

Reviewer comments:

Reviewer #6

(Remarks to the Author)
The authors answered all reviewers' comments appropriately. I recommend publication of this interesting piece of work.

Open Access This Peer Review File is licensed under a Creative Commons Attribution 4.0 International License, which permits use, sharing, adaptation, distribution and reproduction in any medium or format, as long as you give appropriate credit to the original author(s) and the source, provide a link to the Creative Commons license, and indicate if changes were made.
In cases where reviewers are anonymous, credit should be given to 'Anonymous Referee' and the source.
The images or other third party material in this Peer Review File are included in the article's Creative Commons license, unless indicated otherwise in a credit line to the material. If material is not included in the article's Creative Commons license and your intended use is not permitted by statutory regulation or exceeds the permitted use, you will need to obtain permission directly from the copyright holder.
To view a copy of this license, visit https://creativecommons.org/licenses/by/4.0/

We are very appreciative of the reviewers' thoughtful feedback, critiques and suggestions. We have addressed all reviewers' comments in the revised manuscript as described below. For clarity, our responses are numbered, italicized and in blue font. We hope that the reviewers will agree that our changes and additions in response to their suggestions have significantly improved the overall quality and significance of this study. In essence, our study presents an important advance for combating acquired treatment resistance in melanoma patients with progressive disease, for which there is a critical need for new, more effective therapeutic strategies.

REVIEWER COMMENTS

Reviewer #1 (Remarks to the Author); expert in melanoma:

This manuscript presents a number of interesting results but lacks structure; the data is a bit scrambled.

This doesn´t help the fact that, as the authors point out, the role of Bcl-proteins in melanoma response to MAPKi has previously been published. Thus, there is a lack of novelty.

1.1: *We agree with the reviewer that BCL2 proteins have been previously examined in the context of MAPKi responses in melanomas. However, our study is the first concerted effort to evaluate a large number of molecularly characterized PDXs derived from stage IV melanoma patients who had progressed on standard of care treatments and as such do have any other clinically approved treatment options. Further, whereas previous studies demonstrated that inhibiting BCL2 could prevent or delay the onset of resistance in BRAFi-naïve models, our study is the first to show that inhibiting BCL2 is a strategy to overcome resistance in BRAF-mutant tumors that have already progressed on FDA-approved and BRAF and MEK inhibitors and thus have developed acquired resistance. As there remains a critical unmet need for effective strategies for patients who develop resistance to approved targeted therapies, our study and its results represent a clear advance in the field and addresses a critical unmet clinical need.*

There are some fundamental flaws when explaining some of the results. The manuscript should be edited to be more concise, as it is sometimes difficult to follow the thread. This is exemplified in page 3 of the Results section, when describing the data in Figure 3B.

1.2: *Thank you for these suggestions. We have revised our text for easier interpretation of the results in Fig 3B and also reorganized significant portions of the manuscript to remove redundant information and to improve clarity.*

There is quite some data that doesn´t add much to the main point of the study but that occupies significant proportions of the text. For instance, the bottom 2 paragraphs in page 5 of the Results section could be summarize in a couple of sentences within the discussion section.

1.3: *We have edited this section as suggested to remove redundant text.*

The most novel data is found in figure 4C that yet it gets a bit lost among so many less relevant and novel results. For example, data in figures 4E-H do not add anything meaningful to the importance of MCL-1 in melanoma response to MAPKi. Since Mcl-1 had been part of the story form the beginning, why not directly assess its expression in recurrent tumors to introduce the following results overexpressing and/or inhibiting Mcl-1. Again, it shows a lack of focus that doesn´t help the reader to capture the positives of this study.

1.4: *We agree that the most important data in Fig 4 was the previous panels 4C and 4D. Thus, we have reorganized them into Fig 4A and 4B. While we agree that the results from DNAseq data did not indicate a mutation-based resistance to the combination of Dabrafenib+Trametinib+Navitoclax (DTN), this negative result could be of importance to others working on heterogeneity of resistance and hence we feel should still be included in our results (as Fig 4C). As the GSEA analysis of RNAseq data suggested increased oxidative phosphorylation (OxPhos) in resistance (Fig 4D), we have now functionally confirmed the role of OxPhos in the resistant tumors and presented those results in the new Fig 4 E,F and G. Additionally, the RPPA analysis presented in the original Fig 4D showed mTOR as the most significantly overexpressed pathway in resistant tumors, which we earlier showed to be directly responsible for increasing OxPhos which in turn promotes resistance (PMID: 25297634, PMID: 31439581). Hence this is an important inclusion in Fig 4 H and 4I. We have now also included protein expression data of MCL1 in the pre-DTN and post-DTN samples (Fig 4J, 4K), suggesting its role in resistance, which we then validated using functional overexpression analyses in two PDX models (Fig 5G-5J and Fig S6A-6D).*

Similarly, the data presented in Figure 7 regarding cardiotoxicity seems more like the beginning of a new story rather than the end of this one.

1.5: *Cardiotoxicity is a clear limiting factor for clinical development of MCL1i. It is well known that MCL1i inhibit OxPhos, whereas MAPKi activate it. As we are testing the combination of BRAFi+MEKi+MCL1i in our study, it would be important to understand the effect of this combination on cardiotoxicity- particularly if it could worsen it. While the scope of our study does not permit a full-scale investigation of cardiotoxicity, we saw a need to address it at a molecular level in the context of this treatment combination. Thus, we have now performed BH3 profiling and cardiomyocyte electrophysiology function studies to further strengthen our findings in Fig 7, which shows that the BRAFi + MEKi combination could potentially decrease cardiotoxicity of MCL1i. These results are important and would encourage others to also investigate this treatment combination for mitigating cardiotoxicity of MCL1i.*

In summary, the main issue with this manuscript is that almost every figure introduces a new variable, potential mechanism of action or the involvement of a certain pathway. As a result, the manuscripts fails to convincingly address any single question. This study requires some rethinking, structuring to then rewrite a more round, focused story. For starters, I would suggest focusing the study/results on BRAF mutant PDXs.

1.6: *Thank you very much for these observations and suggestions. As the results from the three BRAF-WT PDXs in our study were not contrasting in comparison to the BRAF-mutant PDXs in their response to the combination treatments, we chose to include them as information for researchers working on therapeutic resistance in BRAF-WT melanomas. Notably, while there are no FDA-approved targeted therapy treatments, NCCN guidelines do recommend treatment with single-agent MEKi in melanoma patients with NRAS mutations, BRAF fusions, and BRAF amplifications. Thus, these results are still clinically relevant and we thus we think important to report.*

As described in our above responses, we have significantly restructured our manuscript and included additional results to round off our findings within the scope of our study. We hope that these revisions will increase the significance of our findings that identified BCL2 as a marker of sensitivity to BRAFi+MEKi+BCL2i, and MCL1 as a marker of resistance, using a large number of the most pertinent in vivo models from treatment-progressed patients. Also importantly, while MCL1i can sensitize resistant melanomas dependent on MCL1, cardiotoxicity of MCL1i is a significant problem, and our updated

mechanistic results strengthen our finding that BRAFi+MEKi combination with MCL1i could potentially decrease cardiac toxicity of MCL1i.

Reviewer #2 (Remarks to the Author); expert in melanoma:

This study examines the effect of inhibiting anti-apoptotic proteins in combination with MAPKi in PDX models of melanoma. Effects range from complete tumor growth inhibition to no effect. The authors make good effort in identifying pathways using protein arrays but these experiments do not yield a clear biomarker. The following concerns arise:

1. Navitoclax has been abandoned from clinical development. What is the rational to use it in these experiments?

2.1: *Although the clinically approved BCL2-specific inhibitor, Venetoclax, has clearly benefited hematological cancer patients, relapses are common and mediated by BCL-xL in many instances. This has led to renewed interest in BCL2/BCL-xL inhibitors like Navitoclax, which while still an experimental agent, is promising for treatment of solid cancers (Skwarska & Konopleva, PMID: 37824434). At the time our study was initiated, Navitoclax was being utilized in phase I trial for patients with BRAF-mutant cancers; and ultimately in a randomized phase II trial for targeted therapy-naïve BRAF-mutant metastatic melanoma patients (NCT01989585). Both trials demonstrated safety and feasibility of combining Navitoclax with BRAF and MEK inhibitors, and the phase II trial met its primary endpoint of improved complete response rate (https://ascopubs.org/doi/10.1200/JCO.2023.41.16_suppl.9511, and manuscript under preparation). Even if Navitoclax is subsequently discontinued from clinical development for solid cancers, our identification of a personalized approach with BCL2/BCL-xL inhibition with BRAFi+MEKi supports the clinical relevance of our results, which can be explored in the future with Navitoclax or other BCL2/BCL-xL inhibitors such as Pelcitoclax which is currently in clinical trials for solid cancers.*

2. What is the effect of the combination on immune cells? Immune PDX models have been developed so it is unclear why these are not used.

2.2: *BCL2 and BCL-xL inhibitors are known to induce myelosuppression, primarily neutropenia by Venetoclax and thrombocytopenia by Navitoclax. However, recent studies showed that these inhibitors may improve immune cell function (refs 28 to 31). Additionally, BRAF inhibition is known to improve anti-tumor immune cell function. Preliminary data from a Phase II clinical trial with DTN indicated improved immune cell function in tumors (manuscript under preparation). Based on the reviewer's suggestion, for experimental validation, we have now tested the effect of DTN and DTV on tumor infiltration by CD4+ and CD8+ cells in a humanized mouse model. Our study shows increased tumor infiltration by these immune cells over a period of 21 days of treatments (please see new Figures 3G-H). Due to costs and complications associated with long-term tumor maintenance in humanized mice even with HLA matching, we could not perform a full-fledged tumor growth study to assess the consequence of these effects on tumor growth. An additional limitation is that the immune system in humanized mice is not completely or equally representative of the human immune system. Hence, we are interpreting our results in the context of these limitations, and we hope that additional results from the phase II trial should provide more clarity when that study is published, and in future studies.*

3. How is BCL2/XL regulated by MAPKI and why is it of use to wait for resistance development before adding the anti-apoptotic protein inhibitors?

2.3: *We thank the reviewer for making this point. MAPKi treatment upregulates BIM, which in turn inhibits BCL2 and BCL-xL in cancer cells (PMIDs: 17525735, 17881896, 23532732, 19542438), including in melanoma patients' tumors (PMID: 24983357). In our study, BIM upregulation correlates with response to treatment with the MAPKi+BCL2i/XLii (Fig 3E) and to a lesser extent with MAPKi and single agent BCL2i/XLi(Fig S3K,L).*

Over 80% of melanoma patients treated with BRAFi+MEKi develop resistance. This suggests that anti-apoptosis protein inhibitors could be combined with MAPKi at the beginning of treatment instead of waiting for development of resistance. However, up until our current study of a comprehensive collection of PDXs, there have not been any concerted efforts to identify the molecular features that would be predictive of response to these combinations. Our study has identified such molecular features using the most relevant in vivo melanoma models, which could pave the way for administering these triple combination treatments, potentially in treatment naïve melanomas. Additionally, our phase II clinical trial with DTN in melanoma patients (manuscript under preparation) has tested this treatment combination in MAPKi-treatment naïve patients.

We agree that there is a need to develop front-line strategies to prevent resistance to BRAFi + MEKi. However, the clinical reality is that there is also a critical unmet need for effective therapies for patients who present with acquired resistance to these agents. In addition to a lack of an effective targeted therapy strategy for these patients, there is a lack of clinical trials. We hope that our results will provide a potential new strategy to meet this critical unmet clinical need. We have added this information to the Discussion section.

Reviewer #3 (Remarks to the Author); expert in Bcl2-family proteins:

The manuscript by Vashisht Gopal and colleagues studies the impact of BCL2 inhibition on BRAFi/MEKi treatments in a large collection of molecularly characterized melanoma PDXs and patient samples. The findings indicated that increased BCL-2 protein levels are associated with treatment resistance. Single BCL2 inhibitors (BCL2i) such as Navitoclax or Venetoclax were ineffective against BRAFi+MEKi-resistant PDXs. However, combining BRAFi+MEKi with BCL2i induced potent tumor inhibition and regression, correlated with high basal BCL2 and low basal MCL-1 levels. In contrast, high basal MCL-1 and treatment-induced MCL1 were associated with resistance to BRAFi+MEKi+Venetoclax and BRAFi+MEKi+Navitoclax. Additionally, MEKi treatment counteracted mitochondrial OxPhos inhibition and cardiac troponin activation caused by MCL1i.

The manuscript is well organized. The methods are appropriate, and the results are clearly described and properly discussed.

3.1: *We thank the reviewer for this feedback that the manuscript is well-organized and results well-described.*

References are relevant and quite updated, but a few references are missing. Thus, this study confirms previous articles exploring the potential use of BH3 mimetics (such as venetoclax, navitoclax and

AZD5991) to enhance DT treatment. We have some comments and suggestions that would benefit the manuscript.

Some detailed comments can be found below:

• Conclusions are not new, every PDX/patient responds slightly differently. The authors do not mention the need for biomarkers for personalized medicine.

3.2: *Thank you for this suggestion. The first paragraph of our revised Discussion section now includes a brief explanation of the value of developing a biomarker-guided personalized therapeutic strategy.*

• That the anti-apoptotic BCL-2 family proteins mediate resistance to BRAFi+MEKi in melanoma is not new and has been previously reported by several groups. Please include the appropriate references in the manuscript such as PMID: 31727888, PMID: 31727958, PMID: 37460459.

3.3: *Thank you for pointing this out. PMID: 31727888 was previously included in the section on MCL1-mediated resistance. PMID: 31727958 is now included in the same section. PMID: 37460459 is now included in the introduction.*

• The authors should provide evidence on BCL-xL inhibition, that has not been explored in the manuscript.

3.4: *Thank you for this question. As it is difficult to delineate downstream molecular effects of BCL2 and BCL-xL inhibition, we have performed BH3 profiling of the PDX-derived cells to show the effect of BCL-xL specific peptide, HRK as well as Navitoclax and the BCL-xL specific inhibitor A1331852 (Figures 2H, S3E 6D). For tumor growth inhibition studies, we did not test BCL-xL-specific inhibitors as these are clinically unviable. However, next-generation BCL-xL targeting PROTACS may have the benefit of decreasing platelet toxicity while increasing anti-tumor activity (NCT04886622), and we will explore those in future studies.*

• Is the increase of BIM expression a biomarker for BCL2 inhibition? It has been reported that MAPK inhibition leads to BIM accumulation.

3.5: *Yes, BIM is upregulated by MAPKi as well as BCL2i treatments. The data is shown in Fig 3E, Fig S3K and S3L.*

• It would be interesting if the authors explored the potential patient stratification using BCL2 and BCL-xL protein expression, not only MCL-1.

3.6: *We agree that it would be interesting to use the same stage IV melanoma TMA to perform patient stratification using BCL2 and BCL-xL IHC staining. Unfortunately, we had a limited set of slides of this TMA, and we had previously validated BCL2 and BCL-xL antibodies for IHC using a different set of patient samples. As MCL1 IHC was not previously validated, we used the stage IV TMA to validate a MCL1 antibody for the current study using the stage IV TMA, which also gave us the opportunity to perform melanoma stratification with MCL1 protein (Fig S5A, S5B), and also to quantify MCL1 in pre- and post-DT patient tumors (Fig 5A and 5B). In future studies, we will prioritize stratification of melanoma tumors for all BCL2 family proteins. Until then, the available RPPA data for melanoma TCGA and our large collection of PDXs (Fig 1A and 1B) should prove to be similarly effective for performing such a stratification.*

• In figure 5E the authors should represent MCL-1 increased expression individually, since there is only one clear PDX sample that doubles the expression of this protein.

__3.7:__ Thank you for this suggestion. We understand the rationale for this and have included that data for reviewer's reference (please see slide #2 in the file named "FigureNotShown_Editor&Reviewer_Ref". We did not include this figure in the manuscript as we do not yet have a clear understanding of how much of an increase of MCL1 could mediate resistance. If however the reviewers or editors see a need to include it in the manuscript, we can include it in the supplementary results.

• Have the authors checked in figure 6E if the tumor treated with DT adapts through MCL-1?

__3.8:__ The WM4380_R PDX was established from a tumor biopsy of a patient who developed progressive disease after treatment with Dabrafenib, and it showed very high MCL1 expression. Our hypothesis was that the high MCL1 in this PDX contributed to its resistance to Dabrafenib, and potentially also DT. In this PDX, DT treatment slightly increased MCL1 levels versus vehicle (Fig S6E), and also inhibited tumor growth versus vehicle (Fig S2E), but it did not cause complete tumor regression or stasis. Please note that we defined efficacy as tumor stasis or complete regression, because these are necessary endpoints for clinical benefit. The combination of DT with the MCL1i AZD5991 resulted in complete regression of this PDX (Fig 6E), confirming the role of MCL1 in adapting to/resisting DT treatment.

• BCL family of proteins should be changed to BCL-2 family of proteins throughout the text

__3.9:__ Thank you, this change has been made throughout the manuscript.

• The authors should explore the expression of sensitizer BCL-2 family proteins such as NOXA, BAD and HRK, that directly affect anti-apoptotic proteins pro-survival efficacy.

__3.10:__ As Reviewer #4 had a related question, we performed BH3 profiling with peptides for PUMA, MS1 (instead of NOXA), BAD and HRK, which allowed for exploration of functional roles of these proteins on their effect on the survival of melanoma PDXs. While we did use NOXA in experiments focused on MCL1 (Fig 6D and 7F), our observations and other studies indicate that it was not as effective/sensitive as MS1.

Minor comments:

• Please revise the manuscript and properly reference the figures. Some figures are not well indicated in the text

__3.11:__ Thank you for pointing this out. We have now clearly indicated the figure references in the text throughout.

__**Reviewer #4**__ (Remarks to the Author); expert in Bcl2-family proteins:

The manuscript by Gopal and colleagues reports that combining inhibition of BCL2 family members can increase the response of BRAFV600E-mutant melanoma response to BRAF and MEK inhibition. While a prior phase II clinical trial failed to show any significant improvement of combining navitoclax with BRAF and MEK inhibition, the authors sought to see whether stratifying patient-derived melanoma samples could reveal an impact. Their findings indicate that patients with high levels of BCL2 did show a therapeutic benefit when either venetoclax (V) or navitoclax (N) were added to dabrafenib (D) and

trametinib (T). In some samples, they found that increases in MCL1 expression facilitated resistance. Therefore, they showed using MCL1 overexpression or MCL1 inhibition could render PDX samples resistant or susceptible to D and T respectively. Finally, they argued that MCL1 inhibition, which has been linked to cardiotoxicity in phase I clinical trials, did result in a signaling associated with repression of oxidative phosphorylation and troponin release, but this appeared to be repressed by D and T treatment.

The findings of this study could be therapeutically beneficial in a devastating disease; therefore, the study is of interest. Overall, the study was well performed with rigorous experimentation, data analyses, and robust methodologies including a large panel of PDX samples. Despite these strengths, some weaknesses remain that should be addressed.

1. The manuscript seems to have added the impact on cardiomyocytes to the story at a later date. No methods are provided for any of these experiments. How much of the drugs are being used? What is the source of the human cardiomyocytes? How is troponin IHC being assessed?

4.1: *Thank you for pointing this out. Methodology, source of the human cardiomyocytes and troponin antibodies are now included in the methods section. IHC analysis and H-scores determination are already included in the methods and clarified to include mouse hearts. The concentrations of the drugs used are now indicated in the figure legend of Fig 7.*

2. How can the authors disentangle the impact of combination therapies inducing death versus impacting oxidative phosphorylation in Figure 7? If the impact on oxidative phosphorylation is merely due to the early stages of BAX/BAK-triggered mitochondrial permeabilization (so called MOMP), these data may be misinterpreted.

4.2: *Thank you for this question. We performed BH3 profiling which showed that human cardiomyocytes show selective depolarization with MS1 peptide, suggesting sensitivity to this MCL1-selective sensitizer peptide, and to a smaller extent by BID peptide (Fig 7F). Interestingly, DT treatment decreased depolarization by MS1. Combination of DT with AZD5991 did not further increase depolarization by AZD5991. As mentioned by the reviewer, inhibition of OxPhos by MCL1 inhibition may be triggered by changes in membrane permeabilization. However, OxPhos upregulation by MAPKi is associated with increased mitochondrial fitness and anti-apoptosis rather than short-term alterations in permeabilization, which could counter MCL1i-mediated permeabilization and disruption of cardiomyocyte function.*

3. The response of cancer cells to BCL2 antagonists is often complex and not well predicted by expression alone. In this study, the authors do assess protein levels, which are more reflective of expression than mere mRNA expression. However, even with this it has been a struggle to predict which tumors respond. Thus, many laboratories use a functional assay to predict responses, called BH3 profiling (PMID: 33217342, 34315868, 37210412, etc,). This approach has the benefit of assessing not only the upregulation of anti-apoptotic family members, but also the repression of pro-apoptotic family members. Thus, it might provide a more comprehensive sense of which melanoma samples might be predicted to respond. Also, it might explain why some tumors do not upregulate MCL1 expression, but become resistant to DTV or DTN. Perhaps these samples downmodulate pro-apoptotic molecules. Thus, the overall functional assessment would be that they are less primed to MCL1 inhibitors or peptides.

4.3: *Thank you for this comment and suggestion. Unlike with PDX tumors which facilitated the evaluation of BCL2 family proteins in protein lysates from every PDX in this study, we were unable to isolate functionally viable cells from every PDX for BH3 profiling. This is consistent with melanoma patient tumors from which it is also difficult to isolate cells for in vitro studies. However, we have been able to isolate viable cells from up to eight PDXs. We have performed BH3 profiling in these cells and have presented this data in Fig 2H, Fig S3D-E, Fig 6A-D and Fig 7F-G). For the most part, the levels of BCL2, BCL-xL and MCL1 proteins seem to be suggestive of their functional roles as determined by the BH3 profiling studies. Interestingly, we found that we had to use very high doses of the BH3 peptides and mimetics to induce depolarizations in the PDX-derived melanoma cells versus leukemia cells for example.*

4. A significant portion of the manuscript are linked to impact on gene expression (S4, S7, Figure 7, etc.). While I understand that this allows signatures to be assessed such as impact of oxidative phosphorylation, mTOR, etc. none of these are validated, nor is there any real interpretation of the data. This seems like a missed opportunity.

4.4: *Thank you for making this point. We have now performed Seahorse bioenergetics assays (Fig 4E-G), which validates the role of Oxidative Phosphorylation in the treatment-resistant PDX cells.*

RPPA analysis is a robust and quantitative method to analyze activation status of proteins, which revealed that mTOR was significantly activated in PDXs that were resistant to treatments (Fig 3B, 4H-I) and was significantly downregulated when sensitized (Fig 6H, S6F). While we can use additional methods to prove these effects, we have done this in earlier studies to show the importance of mTOR and OxPhos (PMID: 25297634, PMID: 31439581) in resistance. Rather than repeat the same experiments, we decided to focus on other novel aspects in this study.

Reviewer #5 (Remarks to the Author); ECR:

I co-reviewed this manuscript with one of the reviewers who provided the listed reports. This is part of the Nature Communications initiative to facilitate training in peer review and to provide appropriate recognition for Early Career Researchers who co-review manuscripts.

5. 1: *Thank you for your contribution and the reviewer's support of early career investigators.*

Reviewers' comments:

Reviewer #1 (Remarks to the Author):

We have responded to all of the reviewer's comments labeled 1 through 9.

1. The manuscript has gained some clarity but it is still complicated to follow. The data doesn´t build up into sound, consistent story.

Respectfully, this statement is inconsistent with Reviewer #3's initial review, which stated- ***"The manuscript is well organized. The methods are appropriate, and the results are clearly described and properly discussed."*** *We have, however, provided further clarity on the specific aspects pointed out by reviewer 1. We have also re-organized some of the text and figures to remove any complications in interpretation of results.*

2. Throughout the text, there are too many speculative statements. This is mainly because there is too much variability in tumor response, gene expression correlations etc… and one cannot draw general conclusions. I still find it doesn´t add enough novelty to the field.

We respectfully disagree. Our intent was to interpret results and provide conclusions in a straightforward manner. Any forward-looking statement was made within the context of existing knowledge in the field and was not speculative. We have edited the language in specific instances indicated by the reviewer to provide further clarity. With regards to the comment- "variability in tumor response, gene expression correlations, etc…", such variability is expected in a study like ours which tested a large number of tumors from different patients. It represents real-world patient heterogeneity, and we used rigorous statistical testing to identify significant effects. We have responded to the 'novelty' statement in response to the "Point 9" below.

3. line145- Navitoclax or Venetoclax induced minimal to no growth inhibition of all PDXs in the study. This is just not true, there 6-7 cases where N o V produced obvious anti-tumor effects.

*We agree that there were growth inhibitory effects with N and V, <u>but we used a higher bar to interpret treatment responses</u>. Time and again, we have seen drugs that advance to clinical studies based on statistically significant but small or intermediate tumor growth inhibition in mice, which eventually do not translate to real-world response in patients. As an example in melanoma, BRAF mutant melanoma models treated with Sorafenib achieved statistically significant tumor growth inhibition, but not growth arrest or regression (PMID: 15781657)– and it subsequently failed to achieve clinical benefit in BRAF-mutant metastatic melanoma patients (PMID: 16880785). In contrast, the next-generation BRAF inhibitors (i.e. Vemurafenib, Dabrafenib), achieved complete arrest of tumor growth or regression in vivo in BRAF-mutant melanoma xenografts- and achieved significant clinical responses in treatment-naive patients. Consistent with this, the National Cancer Institute PDXnet Consensus Recommendations for the assessment of anti-tumor activity in PDX models (Meric-Bernstam et al, 2024, PMID: 38641411) state the following: "Small amounts of growth inhibition may be statistically significant with large cohorts. However, if tumor growth stabilization or regression is not achieved, these differences may not be clinically meaningful". Therefore, tumor growth stabilization (<u>**stasis**</u>) or <u>**regression**</u> of tumors were the effects we were*

assessing our treatments for, because such effects can potentially translate to response in patients. We have now changed the text in our revised manuscript for maximal clarity of this interpretation of our tumor growth inhibition results (now page 7, lines 150-161). For additional clarity, we have changed the statistical significance testing from comparing treatment effects on individual days to comparing them over the entire treatment period (Fig 2C-F, Fig S2A-S2M).

4. Lines 153-165. This paragraph is filled with speculative conclusions that are not sufficiently substantiated by the data.

We respectfully disagree. Lines 153 to 155 were simply the definition of BH3 profiling. Lines 156 to 165 were straightforward interpretation of data based on this definition with no speculation. Also please note that the BCL2/BH3 expert Reviewers 3, 4 and 5 did not have a concern with our conclusions of the BH3 experimental results. However, in the interest of the broad reader base of Nature Communications, we have changed the text in the revised manuscript to more clearly explain the BH3 experiment and results (now page 7, line 162, to page 8, line 178). We also added an extra schematic figure of BH3 protein relationships (new Fig S2A) to improve ease of understanding the BH3 profiling results. To further improve interpretation of results from the BH3 profiling heatmaps in Fig 2, we have now added two new figures showing that functional depolarization by PUMA also significantly correlated with treatment induced changes in PUMA protein (Fig S3L) and tumor burden (Fig 3E).

5. Figure 3B. It is really difficult to withdraw such conclusions from the heatmap thereby presented.

We understand this concern. We provided the heatmap to show the results for all of the pathways assessed by the RPPA, for full transparency and for the benefit of readers who may be interested in such results, including pathways that were not significantly associated with outcomes. Our conclusions are based on statistical significance testing. As we describe in the accompanying text, Pearson correlation analysis shows that the Apoptosis pathway is significantly high in the most sensitive PDXs. The most resistant PDXs show significantly high TSC-mTOR and RAS-MAPK pathways, which we have now marked with asterisks for clarity and also clarified that in the text (page 8, line 192, to page 9, line 195). We emphasize again that the observed heterogeneity in protein and gene expression pathways is expected from such a large and diverse collection of patient tumors.

6. line 187- melanoma cells derived from eight PDXs showed a similar correlation of basal BCL2 levels with DTN treatment-induced depolarizations (Fig S3D) but not with BCL-xL levels (Fig S3E), This correlation is not statistically significant.

We agree the result in this supplementary figure is not significant, and we did not say that it is. This result simply suggests that BCL2 protein levels are not fully concordant with BH3 profiles in interpreting responses. This was indicated by Reviewers 3 and 4 in their initial review, when they suggested that we perform BH3 profiling to evaluate the same. In that context, we feel that this result is important to be included at least in the supplementary data. We have however now

adjusted the text describing this observation (page 10, lines 218-232) to better explain these results, which are now in Fig S3M and S3N.

7. Data in figure 3G-H, is not solid enough to draw such definitive conclusions.

We respectfully disagree. For Fig 3G-H, we had provided a straightforward interpretation and conclusion of the results in lines 217 to 222 (now page 10, line 236 to page 11, line 243). The results show that the treatments did not decrease immune cell infiltration in tumors. Some treatments induced significant *increases in tumor infiltration by CD4 and CD8 immune cells, which would suggest a potential beneficial effect. We further clarified that in the text and changed the color of the asterisks from black to red to clearly indicate significant differences.*

8. The differences between WM3690_R-vec and WM3690_R-MCL1 tumors responses to treatments look weak. "Most importantly, compared to -vec tumors (Fig 5I and Fig S6C), -MCL1 tumors were significantly resistant to growth inhibition by single agent N or V or DTN/DTV combination treatments (Fig 5J and S6D), confirming the role of MCL1 in resistance. This sentence is not sustained by the data presented.

We respectfully disagree. In the referenced figures, MCL1 overexpression clearly induced a significant change of tumor growth of the vehicle treatments. Further, in Figure 5I and 5J, MCL1 overexpression changed the DTN treatment response of tumors from complete regression to only significant inhibition. This goes back to our response to "Point 3" above that simply inhibiting tumor growth significantly, as opposed to inducing stasis or regression, may not translate to real world response in patients. We have now added a statement to that effect in the text (page 15, lines 347-356). To further improve interpretation, we have now included area under the curve (AUC) analysis and Tumor Growth Inhibition (TGI) analysis to directly compare treatment effects between empty vector and MCL1 transduced tumors, which show clear signficance of MCL1 overexpression (Figures 5K,L and S6E,F).

9. As suggested in my previous revision, the addition of data on cardiac toxicity doesn´t increase the novelty of the study.

We respectfully disagree with this statement. **A.** *The primary novelty of our study is that it is the first to use a large number of molecularly stratified tumors from melanoma patients who developed resistance to FDA-approved clinical treatments, leading to the identification of biomarkers of complete response to the combination of BRAFi+MEKi with BCL2i/BCL-xLi or MCL1i. Please note that BRAF-mutant melanoma patients who have progressed on standard of care immune therapies and standard of care BRAFi + MEKi regimens have no approved options at this time, and very few clinical trials. Our findings are clinically relevant for such patients who could potentially benefit from this novel predictive biomarker guided treatment.* **B.** *Our study identified for the first time that BRAFi+MEKi treatment may counter the cardiac toxicity of MCL1i. And as far as we know, our study is the first to report the successful use of BH3 profiling to understand cardiac toxicity caused by MCL1 inhibition.*

Reviewer #2 (No remarks to the Authors).

Reviewer #3 (Remarks to the Author):

The authors answered all our comments and the manuscript is now acceptable for publication.

We appreciate the reviewer's recommendation of our study for publication in Nature Communications.

Reviewer #4 (Remarks to the Author):

The revised submission by Gopal and colleagues largely responds to my prior critiques satisfactorily. Most importantly, the revised manuscript provides a revised methods section to explain how the authors used cultured human cardiomyocytes. While these experiments are important, the authors must provide the limitation in the manuscript about the use of IPS-derived cardiomyocyte cultures. Such cultured IPS-derived and differentiated cardiomyocytes are typically most representative of embryonic (that is immature) cardiomyocytes. As a result, these cultured IPS-derived cardiomyocytes typically feature more embryonic metabolism including the utilization of glucose to support oxidative phosphorylation. In contrast, adult cardiomyocytes alter their metabolism to primarily utilize fatty acids to support oxidative phosphorylation. While the authors' studies are not necessarily impacted by this model, it has been reported that MCL1 functions to promote fatty acid oxidation (see Wright et al. Mol Cell 2024). Therefore, models (such as this one) that utilize embryonic cardiomyocyte cultures may not faithfully reflect the toxicities associated with MCL1 inhibition. This limitation should be pointed out in the discussion.

We recognize this limitation that the reviewer has pointed out. We have now included a statement in the discussion that indicates this limitation and potential alternatives to address it in the future (page 21, line 512, to page 22, line 516). We appreciate the reviewer's recommendation of our study for publication in Nature Communications.

Reviewer #5 (Remarks to the Author):

I co-reviewed this manuscript with one of the reviewers who provided the listed reports. This is part of the Nature Communications initiative to facilitate training in peer review and to provide appropriate recognition for Early Career Researchers who co-review manuscripts.

We appreciate the reviewer's mentorship in promoting their junior colleague's participation in reviewing high impact journal manuscripts.
